# National, regional, and provincial prevalence of glaucoma in China in 2020: an updated systematic review and modelling analysis

**DOI:** 10.7189/jogh.15.04268

**Published:** 2025-10-10

**Authors:** Shiyi Shan, Jiali Zhou, Jing Wu, Shanshan Tang, Chenkai Fang, Lingyi Chen, Can Chen, Chenhao Zhang, Igor Rudan, Peige Song, Global Health Epidemiology Research Group (GHERG)

**Affiliations:** 1 Center for Clinical Big Data and Statistics of the Second Affiliated Hospital Zhejiang University School of Medicine, School of Public Health Zhejiang University School of Medicine, Hangzhou, Zhejiang, China; 2 School of Public Health, Zhejiang University School of Medicine, Hangzhou, Zhejiang, China; 3 The Fourth Affiliated Hospital of School of Medicine, and International School of Medicine, International Institutes of Medicine, Zhejiang University, Yiwu, China; 4 Centre for Global Health, Usher Institute, University of Edinburgh, Edinburgh, Scotland, UK; 5 Nuffield Department of Primary Care Health Sciences, Oxford University, UK

## Abstract

**Background:**

Glaucoma is the leading cause of irreversible blindness worldwide, with a significant disease burden in China. Accurate prevalence estimates are crucial for guiding public health strategies, but large-scale epidemiological investigations remain challenging due to the need for comprehensive ophthalmic assessments. Through a systematic review and modelling approach, we aimed to estimate the national, regional, and provincial prevalence of glaucoma in China in 2020.

**Methods:**

We performed an updated systematic literature search of the China National Knowledge Infrastructure, Wanfang, the China Science and Technology Journal Database, PubMed, Embase, and MEDLINE for articles published from 19 August 2017 to 12 July 2024 that reported the prevalence of glaucoma in the general Chinese population. We also included articles from the 2017 China Glaucoma Study whose data collection period started in 2000 or later. We included articles that used optic disc examination and/or visual field assessment for diagnosis, and excluded those defining glaucoma solely by intraocular pressure measurement. We conducted age-sex splitting to enhance data availability for estimating the prevalence of primary open-angle glaucoma (POAG) and primary angle-closure glaucoma (PACG). We applied a multi-level mixed-effects meta-regression to estimate the prevalence of POAG and PACG at national and regional levels, adjusting for age, sex and study year, while we used an associated factor-based model to derive provincial prevalence estimates. We estimated the prevalence of secondary glaucoma and congenital glaucoma through random-effects meta-analyses.

**Results:**

We included 43 articles comprising 286 577 participants. We estimated that the national prevalence of POAG among those aged 20–99 years in 2020 was 0.73% (95% confidence interval (CI) = 0.51, 1.03), equivalent to 7.88 million (95% CI = 5.58, 11.13) affected individuals in the mainland of China. We estimated PACG to be 0.81% (95% CI = 0.60, 1.10), equivalent to 8.79 million (95% CI = 6.50, 11.88) affected population. The prevalence of POAG and PACG both increased with age. The POAG prevalence was higher in males than in females across 20–99 years, while the PACG prevalence was higher in females. The POAG and PACG prevalence varied by region and province; the former was the highest in northeast China (0.77%, 95% CI = 0.55, 1.09) and the lowest in west China (0.71%, 95% CI = 0.50, 1.00), whereas the latter was the highest in central China (0.87%, 95% CI = 0.64, 1.17) and the lowest in east China (0.76%, 95% CI = 0.56, 1.02). Among 31 provinces or municipalities, Shanghai had the highest POAG prevalence (0.80%, 95% CI = 0.56, 1.12) and Tibet showed the lowest (0.56%, 95% CI = 0.40, 0.80). Regarding PACG, Sichuan had the highest prevalence (0.92%, 95% CI = 0.68, 1.25), whereas Beijing exhibited the lowest (0.56%, 95% CI = 0.42, 0.76). The pooled prevalence of secondary glaucoma and congenital glaucoma was 0.22% (95% CI = 0.16, 0.29) and 0.03% (95% CI = 0.00, 0.10), respectively.

**Conclusions:**

The prevalence of glaucoma in China varied significantly across regions and provinces in our study period, highlighting the need for targeted screening programmes, early detection, and resource allocation. The modelling approach used here provides a valuable framework for estimating glaucoma prevalence in settings with limited large-scale epidemiological data.

**Registration:**

PROSPERO: CRD420251005269.

Glaucoma, a progressive optic neuropathy characterised by irreversible optic nerve damage, is the second leading cause of blindness worldwide, disproportionately affecting older adults aged 50 years and above [1]. The condition accounted for 11.0% of global blindness in 2020, with an estimated 3.6 million individuals affected [1]. Unlike cataracts, which are treatable with surgery, glaucoma-related vision loss is permanent [2]. Beyond vision loss, glaucoma significantly impacts individuals’ quality of life, leading to reduced mobility, loss of independence, and decreased productivity, which collectively impose substantial burdens at the individual, societal, and economic levels [3–5].

Glaucoma is often asymptomatic and remains unnoticed until later stages of progression, when significant and irreversible damage has already occurred [6]. Appropriate management can halt or slow the progression of glaucoma, making early screening, diagnosis, and effective treatment essential to prevent further deterioration [2,7]. However, delayed detection remains a major challenge due to the silent nature of glaucoma and the lack of effective screening methods, leading to widespread underdiagnosis and undertreatment, particularly in low-resource settings [7].

Glaucoma has multiple subtypes, with primary open-angle glaucoma (POAG) and primary angle-closure glaucoma (PACG) being the most prevalent. While POAG is the most common form globally, PACG is disproportionately prevalent in Asian populations, including China [8]. Given its large and ageing population, coupled with rapid demographic and lifestyle changes, China faces an escalating glaucoma burden. Our previous systematic review and modelling study of glaucoma prevalence in China, published in 2017, projected that the number of individuals living with glaucoma in China would continue to rise in the coming decades [9]. By 2015, an estimated 13.1 million people in China were living with the condition, with projections indicating this number could rise to more than double by 2050 [9]. The increasing burden of glaucoma is further magnified by the rising prevalence of risk factors for glaucoma, such as myopia [10].

Despite the increasing recognition of glaucoma as a major public health issue, China still lacks comprehensive, accurate, and up-to-date prevalence estimates to inform national and regional health strategies, particularly at the regional and provincial levels. The China National Visual Health Plan (2021–25) highlights the necessity of conducting high-quality epidemiological research on key eye diseases and monitoring the prevalence of major blinding eye conditions in China. Glaucoma, being a leading cause of irreversible blindness, is among the critical eye diseases highlighted in this plan [11]. Addressing glaucoma as a public health priority requires robust epidemiological data to support disease surveillance, healthcare planning, and targeted interventions, particularly as prevalence of the disease continues to increase with population ageing. However, significant gaps in data persist, especially in underdeveloped regions, where diagnostic challenges are compounded by limited access to ophthalmic services [12]. Routine screening methods, such as tonometry and visual acuity assessments, have limited effectiveness in detecting early glaucoma, as intraocular pressure (IOP) alone is insufficient for diagnosis [13], and as visual impairment typically manifests only in advanced stages [6]. Critical assessments, such as optic disc evaluation and visual field testing, are often not available in routine health check-ups, leading to widespread underdiagnosis [14]. As a result, a substantial proportion of glaucoma cases remain undetected, contributing to avoidable blindness across the country [15].

In response to these challenges, in this study, we aimed to provide the most up-to-date and comprehensive estimates of national, regional, and provincial prevalence of glaucoma in China in 2020, using a global health metrics modelling approach. Additionally, we sought to develop a transparent and adaptable methodology for estimating glaucoma prevalence at subnational levels, which could be applied globally in settings with limited ophthalmic resources. Specifically, our intent was toestimate the national prevalence of glaucoma subtypes, including POAG, PACG, secondary glaucoma, and congenital glaucoma; determine the regional and provincial prevalence of primary glaucoma (POAG and PACG); and calculate the total number of individuals affected by POAG and PACG at national and subnational levels in 2020.

## METHODS

We conducted and reported this study in accordance with the PRISMA guidelines and the GATHER statement [16,17]. The systematic review protocol was prospectively registered on PROSPERO (CRD420251005269).

### Systematic review

#### Search strategy and selection criteria

In 2017, we estimated the prevalence of glaucoma in China through a systematic review and modelling analysis [9]. For this study, we performed an updated systematic literature search to identify relevant articles published from 19 August 2017 to 12 July 2024 that reported the prevalence of glaucoma in the general Chinese population, enabling us to generate updated glaucoma prevalence estimates for China. We searched three Chinese and three English bibliographic databases: the China National Knowledge Infrastructure, Wanfang, the China Science and Technology Journal Database (VIP), PubMed, Embase, and MEDLINE. The search strategy combined medical subject headings and free-text terms related to ‛glaucoma’ and ‛prevalence’ (in addition to ‛China’ for three English databases), without language restrictions (Appendix S1 in the **Online Supplementary Document**).

After removing duplicate records, two researchers (ST and CF) independently screened the titles and abstracts of all records, followed by the full texts of any articles remaining at the next review stage. We included articles that were population-based (community- or health check-based); reported the prevalence of glaucoma in the general population across the mainland of China; provided numerical prevalence estimates based on individuals (not eyes); and defined glaucoma using clear diagnostic criteria, including optic disc evaluation and/or visual field assessment, rather than relying solely on IOP measurements. We excluded articles that were hospital-based or conducted in specialised populations (*e.g.* individuals with myopia), or those lacking clear diagnostic methods or reporting self-reported glaucoma prevalence. We also excluded reviews, commentaries, abstracts, case reports, viewpoints, or letters.

Although the included studies used different definitions or examination methods for glaucoma, previous research suggests that the reported prevalence rates were consistent across different definitions [9]. Therefore, our inclusion criteria required that a diagnosis of glaucoma be based on structural or functional indications of glaucomatous optic neuropathy were identified through optic disc examination and/or visual field assessment, rather than relying solely on IOP measurement [18]. For this reason, we included data from the studies that were based on standardised assessments, such as assessments of the optic disc by ophthalmologists using slit-lamp biomicroscopy or fundus photography, and/or visual field testing with automated static perimetry, and/or evaluation of the anterior chamber angle/depth by slit-lamp examination or gonioscopy. All assessments had to be conducted independently of IOP measurement. If multiple publications were based on the same investigation, we retained the most recent study, as it typically included the most up-to-date data. In cases where the study period was identical, we prioritised the publication with the largest data set. If the data sets were also comparable in size, we then selected the publication with the most comprehensive analysis.

To complement our literature search, we retrieved and retained the 26 most recent articles from the 2017 China glaucoma study, including those with study years after 2000 [9]. Additionally, we manually screened the reference lists of all included articles and relevant reviews to identify additional eligible studies.

#### Data extraction and quality assessment

Two researchers (ST and CF) independently extracted data from the included articles, with a third researcher (SS) performing a quality check. We focussed on four major types of glaucoma, namely POAG, PACG, secondary glaucoma, and congenital glaucoma, while extracting relative data on overall glaucoma or four glaucoma subtypes separately. We extracted the following variables into a standardised data collection template:

1. Bibliographic information: title, author(s), publication year, study year, study location, study setting (urban, rural, or mixed), study design, sampling strategy, definition and diagnostic methods of glaucoma (anterior chamber angle/depth evaluation, optic disc evaluation, visual field testing, and IOP measurement).
2. Participant characteristics: sample size, inclusion and exclusion criteria, ethnicity, sex (male, female, or mixed), female proportion of the sample, and age (age range, mean age, median age, or midpoint of the age range).
3. Prevalence estimates: the number of participants who had been tested and the number of people with glaucoma, stratified by age, sex, *etc.*, where available.

For studies that were done in more than one geographical location, we extracted the data for each location separately (if available). We classified the study location into four economic regions according to the economic regional classification in the mainland of China: east China, central China, west China, and northeast China (Appendix S3, Table S1 in the **Online Supplementary Document**). We assessed the quality of included articles using the Quality Assessment Tool, which covers five dimensions: sample population, sample size, participation rate, outcome assessment, and analytical methods [19] (Appendix S3, Table S2 in the **Online Supplementary Document**). The tool is used to assess the overall risk of bias of a study, with quality scores ranging from zero to ten.

We resolved disagreements during the phases of article selection, data extraction, and quality assessment by consensus through discussion with a senior researcher (PS).

### Data imputation

We applied three data imputation assumptions during data extraction. First, we imputed missing data regarding study years as three years before the publication years, based on the average time-lag between publication year and study year derived from the included articles (Appendix S3 and Table S3 in the **Online Supplementary Document**). In case of missing data on age groups, *e.g.* 80 years and above, the missing age band was imputed in two ways. If the average or median age data for the missing age band were absent, we used the same width as other age groups in the same study to impute the missing age band. If the average or median age data for the missing age band were available, we used it to define the missing age band by centring it around the known boundary of the age band. Lastly, we assumed a value of 0.5 for missing data on the proportions of females in a study sample.

To estimate POAG and PACG prevalence in studies that reported only one glaucoma subtype, we applied a meta-ratio approach based on studies that provided concurrent prevalence estimates for both subtypes. First, we identified a subset of studies that reported the prevalence of both POAG and PACG and conducted a random-effects meta-analysis using the DerSimonian and Laird method to calculate the pooled prevalence ratio of POAG to PACG. We then used this ratio to impute missing case numbers for POAG or PACG in studies that reported only one of these conditions. Second, for studies that reported prevalence estimates for primary glaucoma (POAG + PACG) and overall glaucoma, we pooled the ratio of primary glaucoma to overall glaucoma using the same meta-analysis approach. Based on these meta-ratios, we systematically imputed the missing case numbers for primary glaucoma, POAG, and PACG across the respective studies, ensuring a consistent and comprehensive estimation of glaucoma prevalence.

To enhance data availability, we applied an age-sex splitting approach, converting primary extracted data and imputed data for POAG and PACG into age-specific and sex-specific estimates with standardised age and sex groups. To assess the effects of age and sex on POAG and PACG prevalence, we used multilevel mixed-effects meta-regression models. To account for studies that reported zero cases, we applied a continuity correction and replaced zero cells with 0.0005. We incorporated study and province identification as random effects to control for clustering of multiple datapoints within the same study and province. Given that,



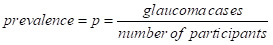



then the prevalence was stabilised by the logit link,



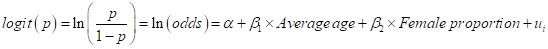



therefore,



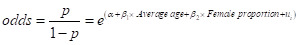



and,



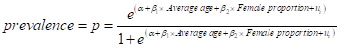



where α is the intercept term, β is the coefficient, and *u_i_* is the random effect.

Based on the above models, we first generated the age-specific and sex-specific prevalence of POAG and PACG (Appendix S3, Figure S1 and S2, and Table S4 in the **Online Supplementary Document**). Datapoints categorised as ‛both’ sexes were divided into male-specific and female-specific estimates, while inconsistent age groups were further split into one-year intervals where necessary.

### Statistical analysis

**Figure 1:**
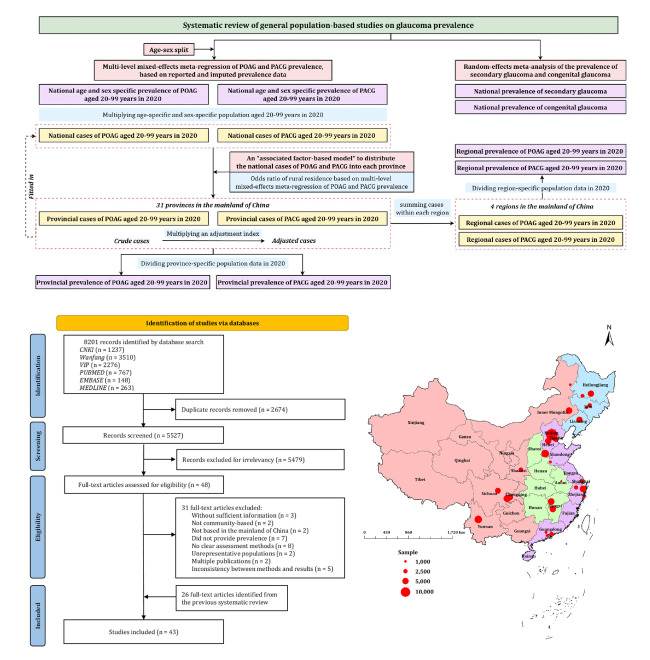
Study flow diagram and location of included studies on glaucoma prevalence in the mainland of China (data from Hong Kong, Macau, and Taiwan were not available). Articles with multiple study locations are not shown in the map. POAG – primary open-angle glaucoma, PACG – primary angle-closure glaucoma.

We estimated the prevalence of each glaucoma subtype (POAG, PACG, secondary glaucoma, and congenital glaucoma) using distinct modelling approaches, tailored to the availability of data for each subtype (**Figure 1**). We then applied multilevel mixed-effects meta-regression models and associated factor-based models to estimate age-sex-specific prevalence at the national, regional, and provincial levels. We restricted the POAG and PACG analysis to individuals aged 20–99 years, where sufficient data were available for model development. For secondary glaucoma and congenital glaucoma, we conducted a random-effect meta-analysis to estimate the national overall prevalence.

#### Epidemiological modelling of national, regional, and provincial prevalence for POAG and PACG

First, based on the age-sex split POAG and PACG prevalence data, we examined the effects of key cluster-level variables, including age, sex, study year, economic region, and study setting, on POAG and PACG prevalence using univariable meta-regression (Appendix S3, Table S5 in the **Online Supplementary Document**). Age, sex, study year, and study setting were found to be significant associated with the prevalence of POAG and PACG. Next, we developed multilevel multivariable mixed-effects meta-regression models, incorporating age, sex, and study year as fixed effects, while study and province identification were included as random effects to account for variability across studies and geographic regions (Appendix S3, Table S6 in the **Online Supplementary Document**). Therefore,



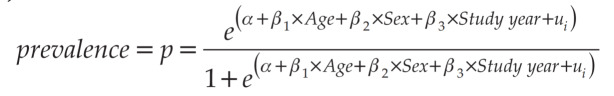



Using these estimates, we calculated the national case numbers (‛national envelopes’) for POAG and PACG among individuals aged 20–99 years in 2020 by multiplying the age- and sex-specific prevalence estimates with the corresponding population data from the 2020 China Census [20]. To account for urbanisation effects, we estimated the odds ratio (OR) of rural *vs.* urban residence, adjusted for age and sex. These adjustments were incorporated into an ‛associated factor-based model’, which we used to distribute the national POAG and PACG case numbers across 31 provinces in mainland China [21]. We then determined the provincial prevalence of POAG and PACG by dividing the estimated number of cases in each province or municipality by its corresponding population. Finally, we aggregated the provincial case numbers to develop regional case estimates (‛regional envelopes’). We calculated the regional prevalence of POAG and PACG by dividing the total number of cases in each region by its corresponding population, ensuring a comprehensive estimation at multiple geographic levels (Appendix S2 in the **Online Supplementary Document****)**.

#### Meta-analysis of the prevalence of secondary glaucoma and congenital glaucoma

We conducted a random-effects (DerSimonian and Laird) meta-analysis to pool the prevalence of secondary glaucoma and congenital glaucoma. Given the rarity of congenital glaucoma and presence of zero values, we stabilised prevalence estimates using the Freeman-Tukey double arcsine transformation. The heterogeneity of the included articles was measured by *I*^2^ index, with *I*^2^ index >50% indicating significant heterogeneity [22]. To examine whether single studies had a disproportionately excessive influence and the robustness of the meta-analysis results, we conducted a leave-one-out sensitivity analysis for each meta-analysis [23]. We detected publication bias by funnel plots, while we performed Egger’s and Begg’s tests for outcomes with ten or more studies.

We conducted all analyses in *R*, version 4.4.2 (R Core Team, Vienna, Austria), and all statistical tests were two-sided.

## RESULTS

We retrieved a total of 8201 records through database searches; 5527 remained after deduplication and 48 were retained for full-text review after title and abstract screening. Of these, 17 articles fit the inclusion criteria. Incorporating an additional 26 records from a previous study with a data collection period starting after the year 2000 [9], 43 articles covering a total of 286 577 participants met the eligibility criteria (**Figure 1**; Appendix S3 and S4, Tables S7–9 in the **Online Supplementary Document**).

Twenty-seven articles (62.8%) reported on the prevalence of POAG, 31 (72.1%) on the prevalence of PACG, 16 (37.2%) on the prevalence of secondary glaucoma, and six (14.0%) on the prevalence of congenital glaucoma. All articles performed optic disc assessments as part of their diagnostic criteria for glaucoma. More than half (n = 33, 76.7%) were published after 2010. All articles had a quality score of five or higher, with the majority scoring eight or higher (38 articles, 88.4%), indicating a high overall quality (Appendix S3, Table S8 in the **Online Supplementary Document**).

We generated three meta-ratios comparing POAG, PACG, primary glaucoma and overall glaucoma (Appendix S3, Figure S3 in the **Online Supplementary Document**), based on which the case numbers for primary glaucoma, POAG, and PACG were imputed. Specifically, the meta-ratio of POAG to primary glaucoma was 0.44 (95% confidence interval (CI) = 0.38, 0.50), that of PACG to primary glaucoma was 0.54 (95% CI = 0.48, 0.60), and that of primary glaucoma to overall glaucoma was 0.89 (95% CI = 0.80, 0.94).

### National, regional, and provincial prevalence and case number of POAG

In the multilevel mixed-effects meta-regression model, age was positively associated with POAG prevalence (β = 0.0255; 95% CI = 0.0251, 0.0258, *P* < 0.0001) (Appendix S3, Table S6 in the **Online Supplementary Document**). We saw a significant difference between sexes, with females having a lower prevalence of POAG compared to males (female: β = −0.4531; 95% CI = −0.4612, −0.4451, *P* < 0.0001). The study year showed a negative association with POAG prevalence (β = −0.0289; 95% CI = −0.0576, −0.0001, *P* < 0.05), suggesting a decrease in POAG prevalence over time.

**Figure 2:**
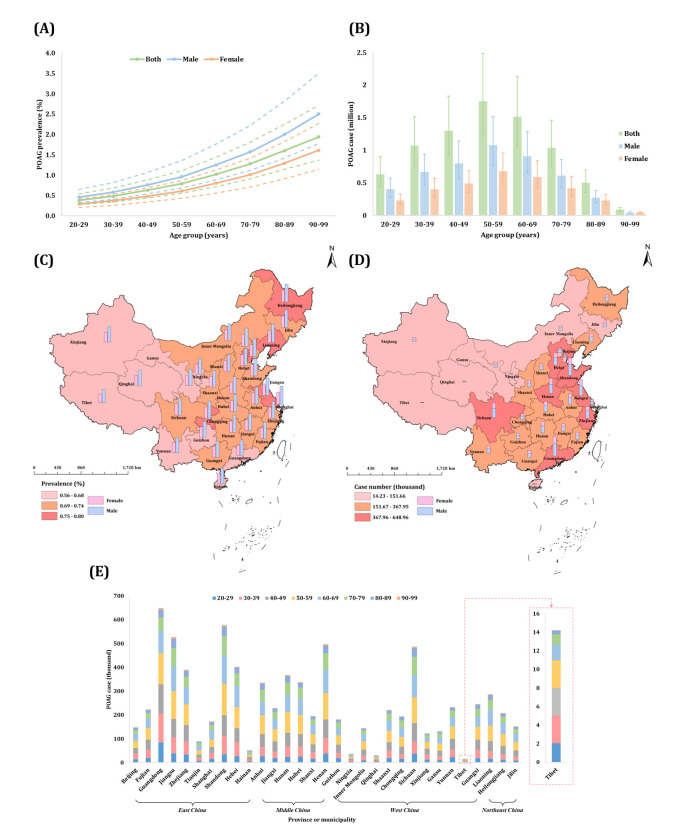
National, regional, and provincial prevalence and case number of primary open-angle glaucoma among individuals aged 20–99 years in the mainland of China in 2020. **Panel A.** National sex-specific prevalence of POAG by age group. **Panel B.** National sex-specific case number of POAG by age group. **Panel C.** National map of POAG prevalence. **Panel D.** National map of POAG case numbers. **Panel E.** Provincial case number of POAG. This study focussed on prevalence in the mainland of China, and data in Hong Kong, Macau, and Taiwan were not available. POAG – primary open-angle glaucoma.

Based on these findings and the availability of corresponding age - and sex-specific population data from the 2020 China Census, we estimated the prevalence for the year 2020 (**Figure 2**, **Table 1**). The overall prevalence of POAG was 0.73% (95% CI = 0.51, 1.03) in 2020. Generally, the prevalence of POAG increased steadily with advanced age, while males exhibited consistently higher prevalence across the whole age spectrum compared to females. A total of 7.88 million (95% CI = 5.58, 11.13) population aged 20–99 years were affected by POAG in mainland China, of whom 60.78% were males (4.79 million, 95% CI = 3.39, 6.76) and 39.22% were females (3.09 million, 95% CI = 2.18, 4.37). We noted a distinct age-specific distribution pattern of case number, with the 50–59 years group exhibiting the highest estimated case count at 1.75 million (95% CI = 1.24, 2.48).

**Table 1:** Estimated sex-specific prevalence and case number of primary open-angle glaucoma in the mainland of China in 2020, by age group

	Primary open-angle glaucoma					
**Age group in years**	**Prevalence, % (95% CI)**			**Case number, million (95% CI)**		
	**Both**	**Male**	**Female**	**Both**	**Male**	**Female**
20–29	0.38 (0.27, 0.54)	0.46 (0.32, 0.65)	0.29 (0.21, 0.41)	0.63 (0.45, 0.90)	0.40 (0.28, 0.57)	0.23 (0.16, 0.33)
30–39	0.48 (0.34, 0.68)	0.58 (0.41, 0.82)	0.37 (0.26, 0.52)	1.07 (0.75, 1.51)	0.67 (0.47, 0.94)	0.40 (0.28, 0.57)
40–49	0.63 (0.44, 0.88)	0.76 (0.54, 1.07)	0.48 (0.34, 0.69)	1.30 (0.92, 1.83)	0.80 (0.57, 1.14)	0.49 (0.35, 0.69)
50–59	0.79 (0.56, 1.11)	0.96 (0.68, 1.36)	0.61 (0.43, 0.87)	1.75 (1.24, 2.48)	1.08 (0.76, 1.52)	0.68 (0.48, 0.96)
60–69	1.02 (0.72, 1.44)	1.25 (0.88, 1.76)	0.80 (0.56, 1.13)	1.51 (1.07, 2.13)	0.91 (0.65, 1.29)	0.59 (0.42, 0.84)
70–79	1.28 (0.91, 1.81)	1.57 (1.12, 2.22)	1.01 (0.71, 1.42)	1.04 (0.73, 1.46)	0.61 (0.43, 0.86)	0.42 (0.30, 0.60)
80–89	1.60 (1.14, 2.25)	2.00 (1.42, 2.82)	1.29 (0.91, 1.82)	0.50 (0.35, 0.70)	0.27 (0.19, 0.38)	0.23 (0.16, 0.32)
90–99	1.94 (1.37, 2.72)	2.50 (1.78, 3.50)	1.61 (1.14, 2.27)	0.09 (0.06, 0.12)	0.04 (0.03, 0.06)	0.05 (0.03, 0.06)
20–99	0.73 (0.51, 1.03)	0.87 (0.62, 1.23)	0.58 (0.41, 0.81)	7.88 (5.58, 11.13)	4.79 (3.39, 6.76)	3.09 (2.18, 4.37)

CI – confidence interval

Across four economic regions, northeast China recorded the highest prevalence of POAG at 0.77% (95% CI = 0.55, 1.09) in 2020, compared to west China’s 0.71% (95% CI = 0.50, 1.00) (Appendix S3, Table S10 in the **Online Supplementary Document**). The case number proportion of east China was the largest, reaching 3.23 million (95% CI = 2.29, 4.56). Marked geographical disparities were evident at the provincial level. Specifically, the highest and lowest prevalence of POAG was observed in Shanghai (0.80%, 95% CI = 0.56, 1.12) and Tibet (0.56%, 95% CI = 0.40, 0.80), respectively. Guangdong had the largest share of national POAG cases (648.95 thousand, 95% CI = 458.99, 916.61), whereas Tibet had the least (14.23 thousand, 95% CI = 10.06, 20.10) (**Figure 2**; Appendix S3, Table S11 in the **Online Supplementary Document**).

### National, regional, and provincial prevalence and case number of PACG

**Figure 3:**
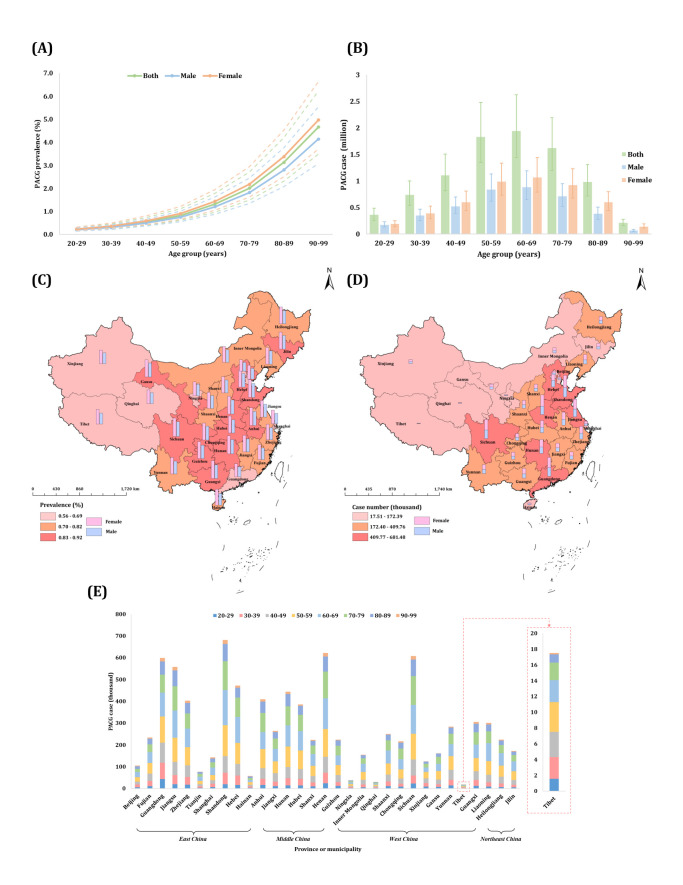
National, regional, and provincial prevalence and case numbers of primary angle-closure glaucoma among individuals aged 20–99 years in the mainland of China in 2020. **Panel A.** National sex-specific prevalence of PACG by age group. **Panel B.** National sex-specific case numbers of PACG by age group. **Panel C.** National map of PACG prevalence. **Panel D.** National map of PACG case number. **Panel E.** Provincial case number of PACG. This study focussed on prevalence in the mainland of China, and data in Hong Kong, Macau, and Taiwan were not available. PACG – primary angle-closure glaucoma.

For PACG, age was positively associated with its prevalence (β = 0.0459; 95% CI = 0.0456, 0.0462, *P* < 0.0001), while females had a higher prevalence compared to males (female: β = 0.1831, 95% CI = 0.1755, 0.1907; *P* < 0.0001). The study year was not significantly associated with PACG prevalence (β = −0.0261; 95% CI = −0.0523, 0.0000, *P* = 0.503), suggesting stability across years (**Figure 3**, **Table 2**; Appendix S3, Table S6 in the **Online Supplementary Document**).

**Table 2:** Estimated sex-specific prevalence and case number of primary angle-closure glaucoma in the mainland of China in 2020, by age group

	Primary angle-closure glaucoma					
**Age group in years**	**Prevalence (%, 95% CI)**			**Case number (million, 95% CI)**		
	**Both**	**Male**	**Female**	**Both**	**Male**	**Female**
20–29	0.21 (0.16, 0.29)	0.20 (0.14, 0.27)	0.24 (0.17, 0.32)	0.36 (0.26, 0.49)	0.17 (0.13, 0.23)	0.19 (0.14, 0.25)
30–39	0.33 (0.24, 0.45)	0.30 (0.22, 0.41)	0.36 (0.27, 0.49)	0.74 (0.54, 1.00)	0.35 (0.25, 0.47)	0.39 (0.29, 0.53)
40–49	0.54 (0.40, 0.73)	0.49 (0.36, 0.66)	0.59 (0.43, 0.80)	1.11 (0.82, 1.51)	0.52 (0.38, 0.70)	0.60 (0.44, 0.81)
50–59	0.82 (0.61, 1.11)	0.75 (0.55, 1.01)	0.90 (0.66, 1.21)	1.83 (1.35, 2.48)	0.84 (0.62, 1.13)	0.99 (0.73, 1.34)
60–69	1.32 (0.97, 1.78)	1.20 (0.88, 1.62)	1.44 (1.06, 1.94)	1.94 (1.44, 2.63)	0.88 (0.65, 1.19)	1.07 (0.79, 1.44)
70–79	2.01 (1.49, 2.71)	1.82 (1.35, 2.45)	2.18 (1.62, 2.94)	1.62 (1.20, 2.19)	0.71 (0.52, 0.95)	0.92 (0.68, 1.23)
80–89	3.13 (2.32, 4.20)	2.80 (2.08, 3.76)	3.38 (2.51, 4.53)	0.98 (0.72, 1.31)	0.38 (0.28, 0.51)	0.60 (0.44, 0.80)
90–99	4.66 (3.48, 6.23)	4.14 (3.08, 5.53)	4.97 (3.71, 6.63)	0.21 (0.16, 0.28)	0.07 (0.05, 0.09)	0.14 (0.11, 0.19)
20–99	0.81 (0.60, 1.10)	0.71 (0.53, 0.96)	0.91 (0.67, 1.23)	8.79 (6.50, 11.88)	3.90 (2.88, 5.28)	4.89 (3.61, 6.60)

CI – confidence interval

We identified a national PACG prevalence of 0.81% (95% CI = 0.60, 1.10). Its age-progressive pattern is more pronounced than that of POAG, with its prevalence in females being slightly higher than that in males across the whole age spectrum from 20 to 99 years. We estimated the total PACG-affected population aged 20–99 years in China to be 8.79 million (95% CI 6.50, 11.88) population in 2020. Of these cases, 44.42% were males (3.90 million, 95% CI = 2.88, 5.28), while 55.58% were females (4.89 million, 95% CI = 3.61, 6.60). Notably, we observed the highest number of estimated cases in the 60–69-year-old age group, with a total of 1.94 million (95% CI = 1.44, 2.63).

We recorded the highest regional prevalence in central China (0.87%, 95% CI = 0.64, 1.17), contrasting with the lowest rate observed in east China (0.76%, 95% CI = 0.56, 1.02). East China exhibited the greatest case number of PACG, reaching 3.33 million (95% CI = 2.46, 4.50) (Appendix S3 and Table S12 in the **Online Supplementary Document**). At the provincial level, we identified Sichuan as having the highest prevalence (0.92%, 95% CI = 0.68, 1.25), whereas Beijing exhibited the lowest prevalence (0.56%, 95% CI = 0.42, 0.76). Shandong accounted for the largest case load (681.48 thousand, 95% CI = 503.86, 920.46) and Tibet bore the smallest proportion (17.50 thousand, 95% CI = 12.93, 23.68) (**Figure 3**; Appendix S3, Table S13 in the **Online Supplementary Document**).

### Prevalence of secondary glaucoma and congenital glaucoma

Sixteen articles reported on the prevalence of secondary glaucoma and six reported on the prevalence of congenital glaucoma. The meta-analysis showed that the pooled prevalence of secondary glaucoma and congenital glaucoma was 0.22% (95% CI = 0.16, 0.29) and 0.03% (95% CI = 0.00, 0.10) (Appendix 3, Figure S4 in the **Online Supplementary Document**). The leave-one-out sensitivity analysis demonstrated that these estimates were robust (Appendix S3, Figure S5 in the **Online Supplementary Document**). We did not detect publication bias in the included studies (Appendix S3, Figure S6 in the **Online Supplementary Document**).

## DISCUSSION

This study provides a comprehensive and updated assessment of the national and subnational prevalence of glaucoma in mainland China in 2020, including estimates for POAG, PACG, secondary glaucoma, and congenital glaucoma. In 2020, the national prevalence of POAG among those aged 20–99 years was 0.73%, affecting approximately 7.88 million individuals; while PACG had a national prevalence of 0.81%, affecting 8.79 million individuals. Age and sex emerged as factors significantly associated with prevalence of both POAG and PACG; specifically, the prevalence of both conditions increased with age, with males showing higher rates of POAG and females exhibiting higher rates of PACG. Regionally, northeast China exhibited the highest prevalence for POAG at 0.77%, while central China had the highest prevalence for PACG at 0.87%. Provincially, POAG prevalence was highest in Shanghai (0.80%) and lowest in Tibet (0.56%), whereas PACG prevalence was highest in Sichuan (0.92%) and lowest in Beijing (0.56%). These findings emphasise the need for targeted glaucoma screening and prevention strategies, particularly in regions with higher prevalence rates, but lower development levels and limited ophthalmic services. Unlike the Institute for Health Metrics and Evaluation approach or World Health Organization estimates, our methodology offers enhanced reproducibility and provides detailed China provincial-level glaucoma subtype prevalence data. We achieved this by coupling a systematic literature update with advanced statistical techniques, including multilevel meta-regression, a meta-ratio approach for data imputation, and an associated factor-based model to distribute national case estimates to the provincial level.

We estimated the prevalence of POAG aged 20–99 years in the mainland of China at 0.73% in 2020, with males showing a higher prevalence than females across all age groups. This finding is consistent with our previous study suggesting that males have a higher risk of developing POAG, especially in older age groups [9]. This is also in line with studies from other populations, where sex differences in glaucoma prevalence have been reported, though the mechanisms underlying these disparities remain complex and multifactorial [24–27]. Differences in risk factors such as hypertension and diabetes between sexes may contribute to these variations [28,29], or they may be partly explained by sex-related anatomical differences [30]. For instance, the higher prevalence of POAG observed in males may be linked to their longer average axial length. Conversely, the higher prevalence of PACG in females could be attributed to their shallower anterior chambers and shorter axial lengths compared to males. The age-related increase in POAG prevalence follows a consistent pattern across both sexes, reinforcing the association between advancing age and the increased risk of glaucoma. This trend suggested efforts to screen for POAG should be targeted towards older populations, particularly males, who appear to be more vulnerable to developing the disease at earlier ages.

For PACG, the national prevalence was slightly higher than POAG at 0.81%, with a pronounced increase with age and among females of all age groups. The higher prevalence of PACG among females is well-documented in the literature and is likely influenced by anatomical differences between sexes, particularly the shallowness of the anterior chamber in females, which predisposes them to angle-closure [30]. Hormonal factors, particularly oestrogen, have also been implicated in influencing the risk of PACG in females, although further research is needed to fully understand the underlying mechanisms [31]. The higher prevalence and severe visual consequences of PACG in females emphasise the need for sex-specific strategies and targeted interventions, such as screening programs for older adults, particularly females.

We noted substantial regional and provincial variations in the prevalence of primary glaucoma, highlighting the geographical heterogeneity within China. Shangai had the highest prevalence of POAG (0.80%), while Tibet had the lowest (0.56%). Shanghai, a highly urbanised region with an aging population, likely has a higher proportion of individuals at risk for POAG due to age and higher rates of risk factors such as hypertension and diabetes. In contrast, Tibet’s lower prevalence may be due to its younger population, with fewer individuals in high-risk age groups.

For PACG, we identified Sichuan as having the highest prevalence (0.92%), whereas Beijing exhibited the lowest (0.56%). For Sichuan, the region’s high-altitude terrain and associated lifestyle factors, such as prolonged exposure to dim lighting and limited access to timely ophthalmic care, may contribute to an increased prevalence [32]. Conversely, Beijing’s advanced healthcare infrastructure, higher health literacy, and comprehensive eye care programmes may improve the management of conditions, such as cataracts and lipid metabolism disorders, which are known risk factors for PACG [33]. These findings underscore the importance of tailoring glaucoma prevention and management strategies to regional characteristics.

Secondary glaucoma, though less prevalent, remains an important component of the overall glaucoma, with an estimated prevalence of 0.22%. Secondary glaucoma arises from various conditions such as ocular trauma, uveitis, or advanced cataracts, and can often be managed if diagnosed early [34]. The relatively low prevalence of secondary glaucoma in our study highlights the need for better awareness of its risk factors, particularly in individuals who may be at higher risk due to comorbid conditions. We found congenital glaucoma to have a prevalence of 0.03%. Despite its low prevalence, congenital glaucoma should not be overlooked, as it can affect eye development and lead to lifetime vision impairment or even vision loss [35]. Timely detection and treatment can improve outcomes and help prevent lifelong visual impairment [35,36]. Therefore, neonatal screening and early referral to ophthalmologists in at-risk populations remains a priority for reducing the incidence of congenital glaucoma-related visual impairment. The robust findings in the sensitivity analysis indicate that these estimates are reliable and provide a solid foundation for future research into the burden of secondary glaucoma and congenital glaucoma in China.

To the best of our knowledge, this is the most up-to-date and comprehensive systematic review and modelling analysis on the national, regional, and provincial prevalence of glaucoma across the mainland of China. The key strengths of this study include its broad coverage of the Chinese population, thorough literature search, and rigorous study selection process. Through an extensive data imputation strategy, we were able to maximise the use of available data, ensuring robust estimates at the national, regional, and provincial levels. To minimise heterogeneity between studies, we focussed on those that assessed glaucoma based on structural or functional evidence of glaucomatous optic neuropathy, rather than relying solely on IOP measurements. This approach has enhanced the detection of early-stage glaucoma. Additionally, we included individuals with POAG across all levels of IOP in our study, providing a more inclusive estimate of prevalence. Importantly, this is the first study to estimate the prevalence of congenital glaucoma in China, offering new insights into both domestic and global glaucoma epidemiology.

Despite these strengths, several limitations should be noted. First, the heterogeneity across the included studies, due to variations in study design, target populations, methods, and settings, may have introduced some degree of bias. Second, we did not exclude studies based on consensus criteria for glaucoma definitions and grading systems but rather relied on the methods used in the individual studies. While previous research suggests that surveys with different definitions and methods provide comparable prevalence estimates, the inherent subjectivity in interpreting ophthalmic images could still influence the results [18]. Third, secondary glaucoma and congenital glaucoma were underrepresented in the existing epidemiological literature. Despite our extensive search strategy, the limited number of studies reporting on these two subtypes, along with the high heterogeneity in prevalence estimates, may affect the robustness of the findings for these conditions. Fourth, limited data were available on the prevalence for certain provinces, particularly the western ones such as Tibet and Xinjiang, which could have affected the precision of our estimates. Future research could focus on expanding the data collection efforts to include more comprehensive and up-to-date prevalence data from underrepresented regions. Finally, we derived the provincial prevalence estimates using a model that considered only age, sex, study year, and setting, potentially overlooking other relevant factors that may influence regional variations in glaucoma prevalence, such as myopia. Future studies incorporating a broader set of factors could provide more accurate and comprehensive estimates of glaucoma prevalence across China.

Our findings provide insights into the epidemiology of glaucoma in China and could have implications for public health and policy. By offering comprehensive prevalence estimates at national, regional, and provincial levels, our research underscores the need for targeted interventions to mitigate the burden of glaucoma. Understanding its geographical variability is essential for guiding resource allocation and developing region-specific prevention and management strategies. To address the challenges posed by the limited availability of ophthalmic services in many developing countries, including China, we have established a transparent and adaptable methodology for estimating glaucoma prevalence at subnational levels, which integrated an updated systematic review with advanced statistical modeling (including multilevel meta-regression and an associated factor-based model) to synthesise the available evidence. This approach can be applied globally, enabling underdeveloped regions to better understand and address the burden of glaucoma in their populations. Future studies are needed to facilitate accurate cross-country comparisons, especially in low- and middle-income countries, and enhance the understanding of glaucoma’s global burden. Despite these advancements, considerable gaps remain in the treatment, monitoring, and intervention of glaucoma in China. Accurate assessment is hindered by limited access to diagnostic tools, disparities in healthcare infrastructure, and the under-recognition of early-stage glaucoma. These challenges are compounded by a scarcity of trained ophthalmic professionals and low public awareness, particularly in rural and underserved areas. To bridge these gaps, targeted interventions are needed to improve early detection and management of glaucoma, especially in high-prevalence and limited resources areas. Our findings provide valuable guidance for policymakers and healthcare providers to design effective, evidence-based interventions, educational initiatives and public health policies aimed at reducing the burden of glaucoma. In particular, the emphasis on cost-effective screening and targeted interventions should be incorporated into policy frameworks to ensure that resources are allocated efficiently and effectively. Ultimately, such efforts have the potential to improve eye health outcomes in China and globally.

## CONCLUSIONS

This is the first comprehensive assessment of glaucoma prevalence across the mainland of China, revealing substantial regional and provincial disparities and a considerable public health burden, necessitating targeted, region-specific interventions. The novel methodology developed here offers a practical framework for estimating glaucoma prevalence in contexts lacking nationwide screening data, with potential applicability in other countries. These findings can inform evidence-based strategies to reduce the burden of glaucoma and improve eye health outcomes in China and worldwide.

## Additional material


Online Supplementary Document
