# Supplementary material for: National, regional, and provincial prevalence of glaucoma in China in 2020: an updated systematic review and modelling analysis
Source: J Glob Health. 2025 Oct 10;15:04268. doi: 10.7189/jogh.15.04268 (PMC12512002; doi:10.7189/jogh.15.04268)
Supplement: Online Supplementary Document [file jogh-15-04268-s001.pdf]

Supplement to: Shan S, Zhou J, Wu J, Tang S, Fang C, Chen L, Chen C, Zhang C, Song P, Rudan I. National, regional, and provincial prevalence of glaucoma in China in 2020: an updated systematic review and modelling analysis. J Glob Health. 2025;15:04268.

Supplementary Appendix

Shiyi Shan[1,2], Jiali Zhou[1,2], Jing Wu[1,2], Shanshan Tang[1,3], Chenkai Fang[1,2], Lingyi Chen[1,2], Can Chen[1,2], Chenhao Zhang[1,2], Igor Rudan[4,5]*, Peige Song[1,2,4]*; on behalf of the Global Health Epidemiology Research Group (GHERG)

[1]Center for Clinical Big Data and Statistics of the Second Affiliated Hospital Zhejiang University School of Medicine, School of Public Health Zhejiang University School of Medicine,Hangzhou, Zhejiang, China
[2] School of Public Health, Zhejiang University School of Medicine,Hangzhou, Zhejiang, China
[3]The Fourth Affiliated Hospital of School of Medicine, and International School of Medicine, International Institutes of Medicine, Zhejiang University, Yiwu, China
[4]Centre for Global Health, Usher Institute, University of Edinburgh, Edinburgh, Scotland, UK
[5]Nuffield Department of Primary Care Health Sciences, Oxford University, UK

*Joint senior authorship.

Corresponding Author: Peige Song, PhD, Center for Clinical Big Data and Statistics of the Second Affiliated Hospital Zhejiang University School of Medicine, School of Public Health Zhejiang University School of Medicine, China (peigesong@zju.edu.cn).
Tel: +86(0)571-88981368

Content

Appendix 1. Search strategy to identify studies reporting the prevalence of glaucoma in China.....3
Appendix 2. eMethods: The detailed description of stages used to derive national, regional, and provincial estimations of the prevalence of primary open-angle glaucoma and primary angle-closure glaucoma.....5
Appendix 3. Supplementary tables and figures.....7
Table S1. Four economic regions in the mainland of China.....7
Table S2. Quality assessment scale for rating the risk of bias.....8
Table S3. The time-lag between study and publication in the included articles.....9
Table S4. Multilevel mixed-effects meta-regression models for rate patterns of the prevalence of primary open-angle glaucoma and primary angle-clousure glaucoma.....11
Table S5. Univariate meta-regression models of the prevalence of primary open-angle glaucoma and primary angle-clousure glaucoma.....12
Table S6. Multilevel mixed-effects meta-regression models of the national prevalence of primary open-angle glaucoma and primary angle-clousure glaucoma.....13
Table S7. Detailed characteristics of the included articles (n=43).....14
Table S8. Quality scores for assessing the risk of bias in the included articles (n=43).....19
Table S9. Main characteristics of the included articles (n=43).....21
Table S10. Estimated age- and sex-specific prevalence and case number of primary open-angle glaucoma by economic regions in the mainland of China in 2020.....22
Table S11. Estimated provincial prevalence and case number of primary open-angle glaucoma in the mainland of China in 2020.....24
Table S12. Estimated age- and sex-specific prevalence and case number of primary angle-closure glaucoma by economic regions in the mainland of China in 2020.....26
Table S13. Estimated provincial prevalence and case number of primary angle-closure glaucoma in the mainland of China in 2020.....28
Figure S1. Rate pattern for primary open-angle glaucoma.....30
Figure S2. Rate pattern for primary angle-closure glaucoma.....31
Figure S3. The meta-ratio of primary open-angle glaucoma to primary glaucoma, primary angle-closure glaucoma to primary glaucoma, and primary glaucoma to overall glaucoma..32
Figure S4. The pooled prevalence of secondary glaucoma and congenital glaucoma in the mainland of China.....33
Figure S5. Leave-one-out sensitivity analysis for secondary glaucoma and congenital glaucoma.....34
Figure S6. Funnel plots for the prevalence of secondary glaucoma and congenital glaucoma.....35
Appendix 4. Full list of the included articles (n=43).....36

Appendix 1. Search strategy to identify studies reporting the prevalence of glaucoma in China

Database	Access date	Subject category	Sub-database	Search terms	Publication date	Search method
**CNKI**	31/7/2024	Medicine & Public Health	Journal, Featured journal, Doctoral dissertation, Master dissertation	(SU%='青光眼') AND (SU%='发病率'+'发生率'+'罹患率'+'患病率'+'现患率'+'死亡率'+'病死率'+'流行'+'负担'+'现况调查'+'现况研究')	19/8/2017-31/7/2024	Comprehensive search: subject, title, keywords and abstract
**Wanfang**	31/7/2024	Not applicable	Journal article, Dissertations	((主题:(青光眼)) and (主题:(发病率) or 主题:(发生率) or 主题:(患病率) or 主题:(罹患率) or 主题:(现患率) or 主题:(死亡率) or 主题:(病死率) or 主题:(流行) or 主题:(负担) or 主题:(现况调查) or 主题:(现况研究))) and 发表时间:2017-*	2017-2024	Comprehensive search: subject (including title, keywords and abstract)
**VIP**	31/7/2024	Medicine & Public Health	All journals	(M=(青光眼) OR R=(青光眼)) AND (M=(发病率 OR 发生率 OR 患病率 OR 罹患率 OR 现患率 OR 死亡率 OR 病死率 OR 流行 OR 负担 OR 现况研究 OR 现况调查) OR R=(发病率 OR 发生率 OR 患病率 OR 罹患率 OR 现患率 OR 死亡率 OR 病死率 OR 流行 OR 负担 OR 现况研究 OR 现况调查))	2017-2024	Comprehensive search: subject, title, keywords and abstract
**PubMed**	31/7/2024	Not applicable	Not applicable	((Glaucoma) AND (China OR Chinese OR Hongkong OR Macao OR Taiwan) AND (inciden* OR prevalen* OR morbidity OR mortality OR epidemiology)) AND ("2017/08/19"[Date - Publication]: "2024/07/31"[Date - Publication])	19/8/2017-31/7/2024	Comprehensive search: all fields
**Embase**	31/7/2024	Not applicable	Not applicable	#1<br>'glaucoma'/exp OR 'glaucoma':ab,ti<br>#2<br>('China'/exp OR 'China':ab,ti) OR ('Chinese'/exp OR 'Chinese':ab,ti) OR ('Hong Kong'/exp OR 'Hong Kong':ab,ti) OR ('Macao'/exp OR 'Macao':ab,ti) OR ('Taiwan'/exp OR 'Taiwan':ab,ti)<br>#3<br>('incidence'/exp OR 'inciden*':ab,ti) OR ('prevalence'/exp OR 'prevalen*':ab,ti) OR ('morbidity'/exp OR 'morbidity':ab,ti) OR ('mortality'/exp OR 'mortality':ab,ti) OR ('epidemiology'/exp OR 'epidemiology':ab,ti)<br>#4<br>'#1 AND #2 AND #3<br>#5<br>#4 AND [embase]/lim NOT ([embase]/lim AND [medline]/lim) AND [2017-2024]/py	01/01/2017-31/7/2024	Comprehensive search: all fields
**MEDLINE**	31/7/2024	Not applicable	Not applicable	#1<br>exp Glaucoma/ OR (glaucoma).ab,ti.<br>#2<br>exp China/ OR exp East Asian People/ OR exp Hong Kong/ OR exp Macau/ OR exp Taiwan/ OR (China or Chinese or Hong Kong or Macao or Taiwan).ab,ti<br>#3<br>exp Incidence/ OR exp Prevalence/ OR exp Morbidity/ OR exp Mortality/ OR exp	01/01/2017-31/7/2024	Comprehensive search: all fields

Database	Access date	Subject category	Sub-database	Search terms	Publication date	Search method
				Epidemiology/ OR (inciden* or prevalen* or morbidity or mortality or epidemiology).ab,ti.<br>#4<br>1 and 2 and 3<br>#5<br>limit 4 to yr="2017 -Current"		

Notes: Number of records returned was 8,201. The access date was 31 July 2024.

Appendix 2. eMethods: The detailed description of stages used to derive national, regional, and provincial estimations of the prevalence of primary open-angle glaucoma and primary angle-closure glaucoma

This section is a supplement to the Methods part in the main text.

Stage 1 Age-sex splitting for primary open-angle glaucoma (POAG) and primary angle-closure glaucoma (PACG)

Stage 1.1 Age- and sex- specific prevalence pattern for POAG and PACG

Multiple data points specifying age-sex-specific prevalence for POAG and PACG were extracted from the included articles. The following analysis was restricted to the 20-99 year age range, where sufficient data were available for model development. To provide a basis of age-sex splitting for POAG and PACG, we first adopted multilevel mixed-effects meta-regression models to explore the effects of age and sex on the prevalence of POAG and PACG. To enable the inclusion of zero cases as reported, zero cells were replaced with a value of 0.0005. The effect of datapoints clustering from the same study and the same province was controlled by adding study and province identification into the regression model as the random effect. Given that,

$$\boldsymbol{prevalence = p = \frac{glaucoma\ cases}{number\ of\ participants}}$$

Then the prevalence was stabilized by the logit link, and established as a function of average age and female proportion,

$$\boldsymbol{logit(p) = \ln\left(\frac{p}{1-p}\right) = \ln(odds) = \alpha + \beta_1 * Average\ age + \beta_2 * Female\ proportion + u_i}$$

Therefore,

$$\boldsymbol{odds = \frac{p}{1-p} = e^{(\alpha+\beta_1*Average\ age+\beta_2*Female\ proportion+u_i)}}$$

And,

$$\boldsymbol{prevalence = p = \frac{e^{(\alpha+\beta_1*Average\ age+\beta_2*Female\ proportion+u_i)}}{1+e^{(\alpha+\beta_1*Average\ age+\beta_2*Female\ proportion+u_i)}}}$$

where $\boldsymbol{\alpha}$ is the intercept term, $\boldsymbol{\beta}$ is the coefficient, and $\boldsymbol{u_i}$ is the random-effect.

Based on the above models, the "prevalence patterns" for POAG and PACG were generated (**Table S3** and **Figure S1-S2**).

Stage 1.2 Age- and sex- specific prevalence pattern for POAG and PACG

Based on the "prevalence patterns" for POAG and PACG, we used an age-sex splitting approach to convert data with standard age and sex groups. Primary extracted and imputed datapoints were first combined, followed by sex split of datapoints specified as "both" sex into male- and female-specific datapoints. Subsequently, datapoints with inconsistent age groups were split into uniform one-year age groups. The following equation was employed for this process:

$$\boldsymbol{C_{a,s} = R_{a,s}N_{a,s}\frac{C_{A,S}}{\sum_{a\in A,s\in S} R_{a,s}N_{a,s}}}$$

In this equation, $\boldsymbol{a}$ is a one-year age group, $\boldsymbol{s}$ is the specific sex (male or female), $\boldsymbol{A}$ is the set of ages the data is aggregated across, $\boldsymbol{S}$ is the set of sexes data is aggregated across, $\boldsymbol{C_{A,S}}$ is the total case number in ages $\boldsymbol{A}$ and sexes $\boldsymbol{S}$ to be split reported, $\boldsymbol{R_{a,s}}$ is the prevalence in age group $\boldsymbol{a}$ and sex $\boldsymbol{s}$ from "prevalence patterns", $\boldsymbol{N_{a,s}}$ is the population in age group $\boldsymbol{a}$ and sex $\boldsymbol{s}$ based on the 2020 population census of China, and $\boldsymbol{C_{a,s}}$ is the split case number of glaucoma in age group $\boldsymbol{a}$ and sex $\boldsymbol{s}$.

After performing age-sex splitting, the case numbers for POAG and PACG from various articles were divided into single-year age groups and separated by sex (male and female).

Stage 2: Epidemiological modelling of national, regional, and provincial prevalence and case number for POAG and PACG

Stage 2.1 Age- and sex- specific prevalence of POAG and PACG at national levels in 2020

To systematically address heterogeneity across studies and hierarchical data structures, multilevel mixed-effects meta-regression models were adopted, based on age-sex split POAG and PACG data. We stabilized the prevalence by the logit link. Thus,

$$\boldsymbol{prevalence = p = \frac{e^{(\alpha+\beta_1*x_1+\beta_2*x_2+\ldots\beta_n*x_n+u_i)}}{1+e^{(\alpha+\beta_1*x_1+\beta_2*x_2+\ldots\beta_n*x_n+u_i)}}}$$

where $\boldsymbol{\alpha}$ is the intercept term, $\boldsymbol{\beta}$ is the coefficient, $\boldsymbol{u_i}$ is the random-effect, and $\boldsymbol{x}$ is the variable.

The effects of several cluster-level variables on POAG and PACG were first assessed independently, including age, sex, study year, economic region, and setting (**Table S4**). To estimate national prevalence of POAG and PACG in 2020 among people aged 20-99 years, we fitted multilevel multivariable mixed-effects meta-regression models with age, sex, and study year as fixed-effect variables, and study and province identification as the random-effects, respectively (**Table S5**). Therefore,

$$\boldsymbol{prevalence = p} = \frac{e^{(\alpha+\beta_1*Age+\beta_2*Sex+\beta_3*Study\ year+u_i)}}{\boldsymbol{1+e^{(\alpha+\beta_1*Age+\beta_2*Sex+\beta_3*Study\ year+u_i)}}}$$

where $\boldsymbol{\alpha}$ is the intercept term, $\boldsymbol{\beta}$ is the coefficient, and $\boldsymbol{u_i}$ is the random-effect.

Based on the above models , the age-, sex-, and year-specific prevalence of POAG and PACG was generated for the mainland of China. The national case number of POAG and PACG aged 20-99 years in 2020 were then generated by multiplying the estimated age- and sex-specific prevalence of POAG and PACG in 2020 with the corresponding population data, obtained from the 2020 population census of China. This process established "national envelopes", representing the total number of POAG and PACG cases across the mainland of China.

Stage 2.2 Age- and sex- specific prevalence of POAG and PACG at provincial levels in 2020

The national case number of POAG and PACG aged 20-99 years in 2020 was then distributed into 31 provinces in the mainland of China through an associated factor-based model, which was initially proposed by Global Health Epidemiology Reference Group and has been widely used in estimating burden of disease at national, regional, and provincial levels. We generated the odds ratio (OR) and 95% confidence interval (CI) of rural residence compared with urban from a multilevel mixed-effects meta-regression with age, sex, and setting as fixed-effect variables, and study and province identification as the random-effects. The OR was estimated as 0.65 (95% CI 0.63, 0.70) for POAG and 1.68 (95% CI 1.63, 1.80) for PACG. The provincial ratio of rural residents aged 20-99 years was obtained from the 2020 population census of China. Then, the provincial numbers of POAG and PACG cases ("province envelopes") were imputed by the following formula:

$$\boldsymbol{N_{province} = POP_{province} * Prev_{glaucoma_{nation}} * (1 + (Ratio_{setting_{province}} - Ratio_{setting_{nation}}) * (OR_{setting} - 1))}$$

Where $\boldsymbol{N_{province}}$ and $\boldsymbol{POP_{province}}$ are the number of POAG and PACG cases and population size aged 20-99 years in each province or municipality. $\boldsymbol{Prev_{glaucoma_{nation}}}$ indicates the estimated national prevalence of POAG and PACG. $\boldsymbol{Ratio_{setting_{province}}}$ and $\boldsymbol{Ratio_{setting_{nation}}}$ are the ratio of rural residents in each province or municipality in the mainland of China. $\boldsymbol{OR_{setting}}$ is the synthesized OR of rural residence compared with urban.

For each province, an "adjustment index" was used to ensure that the sum of provincial cases fit within "national envelopes". Then, the adjusted provincial prevalence of POAG and PACG were calculated by the number of POAG or PACG cases in each province or municipality divided by its corresponding population.

Stage 2.3 Age- and sex- specific prevalence of POAG and PACG at regional levels in 2020

Finally, we developed "regional envelopes" for POAG and PACG respectively, by summing the cases within each region (east China, central China, west China, and northeast China), and calculated prevalence of POAG or PACG by the number of POAG or PACG cases in each province or municipality divided by its corresponding population.

All analyses and visualizations were conducted in R version 4.4.2 (https://www.r-project.org). All statistical tests were two-sided, and *P*-values<0.05 were considered statistically significant.

Appendix 3. Supplementary tables and figures

Table S1. Four economic regions in the mainland of China

Region	Included provinces
East China	Beijing Municipality, Tianjin Municipality, Hebei province, Shanghai Municipality, Jiangsu province, Zhejiang province, Fujian province, Shandong province, Guangdong province, Hainan province
Central China	Shanxi province, Anhui province, Jiangxi province, Henan province, Hubei province, Hunan province
West China	Inner Mongolia Autonomous Region, Guangxi Zhuang Autonomous Region, Chongqing Municipality, Sichuan province, Guizhou province, Yunnan province, Tibet Autonomous Region, Shaanxi province, Gansu province, Qinghai province, Ningxia Hui Autonomous Region, Xinjiang Uyghur Autonomous Region
Northeast China	Liaoning province, Jilin province, Heilongjiang province

Table S2. Quality assessment scale for rating the risk of bias

Bias type	Low risk (score=2)	Moderate risk (score=1)	High risk (score=0)
Selection (sample population)	(1) Sample from general population, not a select group;<br>(2) Consecutive unselected population;<br>(3) Rationale for case and control selection explained.	(1) Sample selected from large population but selection criteria not defined;<br>(2) Sample selection ambiguous but may be representative;<br>(3) Rationale for cases and controls not explained;<br>(4) Eligibility criteria not explained;<br>(5) Analysis to adjust for sampling strategy bias.	(1) Highly select population making it difficult to generalise finding;<br>(2) Sample selection ambiguous and sample unlikely to be representative.
Selection (sample size)	(1) Sample size calculation performed and adequate.	(1) Sample size calculation performed and reasons for not meeting sample size given;<br>(2) Sample size calculation not performed but all eligible persons studied.	(1) Sample size estimation unclear or only sub-sample studied.
Selection (participation rate)	(1) High response rate (>85%).	(1) Moderate response rate (70-85%).	(1) Low response rate (<70%);<br>(2) Response rate not reported.
Performance bias (outcome assessment)	(1) Diagnosis using consistent criteria and direct examination.	(1) Assessment from administrative database or register;<br>(2) Assessment from hospital record or interviewer.	(1) Assessment from non-validated data or generic estimate from the overall population.
Performance bias (analytical methods to control for bias)	(1) Analysis appropriate for the type of sample (subgroup analysis/regression etc.).	(1) Analysis does not account for common adjustment.	(1) Data confusing.

Table S3. The time-lag between study and publication in the included articles

ID	Author (publication year)	Study Year	Time-lag (year)
**S-01**	Xu L et al. (2004)	2001	3
**S-02**	Bai ZL et al. (2005)	2003	2
**S-03**	Xu L et al. (2005)	2001	4
**S-04**	Sun HM et al. (2005)	2003	2
**S-05**	Ren BC et al. (2005)	2003	2
**S-06**	He M et al. (2006)	2003	3
**S-07**	Bai YQ et al. (2007)	2006	1
**S-08**	Yuan HP et al. (2007)	2004	3
**S-09**	Deng ZF et al. (2008)	NA	NA
**S-10**	Song SF et al. (2009)	2005	4
**S-11**	Zhao X et al. (2010)	2006	4
**S-12**	Zhang LJ et al. (2010)	2009	1
**S-13**	Wang YX et al. (2010)	2001	9
**S-14**	Liang YB et al. (2011)	2007	4
**S-15**	Liang Y et al. (2011)	2007	4
**S-16**	Qu W et al. (2011)	2007	4
**S-17**	Song W et al. (2011)	2009	2
**S-18**	Sun J et al. (2012)	2007	5
**S-19**	Zhong H et al. (2012)	2010	2
**S-20**	Yu YY et al. (2013)	NA	NA
**S-21**	Sheng WD (2014)	2013	1
**S-22**	Yin LR et al. (2014)	2010	4

ID	Author (publication year)	Study Year	Time-lag (year)
**S-23**	Gao ZZ et al. (2015)	2011	4
**S-24**	Pan YJ (2015)	2014	1
**S-25**	He J et al. (2015)	2011	4
**S-26**	Pan CW et al. (2016)	2010	6
**S-27**	Cai Ji-wu et al. (2018)	2017	1
**S-28**	ZHU Dan et al. (2018)	NA	NA
**S-29**	ZHANG Min et al. (2018)	2017	1
**S-30**	Jonas J.B. et al. (2018)	2011	7
**S-31**	ZHOU Wei, et al. (2018)	2016	2
**S-32**	Hai-ming Xu, et al. (2018)	2015	3
**S-33**	WU Xiao-lan et al. (2019)	2015	4
**S-34**	LI Tai-dong, et al. (2019)	2018	1
**S-35**	LI Wei-wei, et al. (2019)	2018	1
**S-36**	LIU Yan-jun (2020)	2019	1
**S-37**	HUANG Bo-shu (2020)	2018	2
**S-38**	CHENG hong et al. (2021)	2017	4
**S-39**	Yang, X., et al. (2021)	2018	3
**S-40**	Yan, X., et al. (2021)	2018	3
**S-41**	Yuanyuan, L., et al. (2022)	2018	4
**S-42**	Zuo-xin Qin (2023)	2018	5
**S-43**	Zhang, A., et al (2023)	2018	5

Note: The average time-lag between study and publication was 3.12 based on 40 articles with available data.

Table S4. Multilevel mixed-effects meta-regression models for rate patterns of the prevalence of primary open-angle glaucoma and primary angle-clousure glaucoma

Variable	Number of data points	*β* (95% CI)	*P*-value	I^2 (%)
**POAG**				
Intercept	129	-5.6886 (-6.1072, -5.2701)	**<0.0001**	
Average age	129	0.0241 (0.0188. 0.0299)	**<0.0001**	**80.59**
Female proportion	129	-0.3038 (-0.4226, -0.1850)	**<0.0001**	
**PACG**				
Intercept	155	-7.2067 (-7.5975, 6.8158)	**<0.0001**	
Average age	155	0.0446 (0.0393, 0.0499)	**<0.0001**	**65.87**
Female proportion	155	0.3455 (0.2215, 0.4694)	**<0.0001**	

Notes: CI, confidence interval; POAG, primary open-angle glaucoma; PACG, primary angle-closure glaucoma.

Table S5. Univariate meta-regression models of the prevalence of primary open-angle glaucoma and primary angle-clousure glaucoma

Variable	Number of data points	*β* (95% CI)	*P*-value	I^2 (%)
**POAG**				
Age	3895	0.0250 (0.0247, 0.0254)	**<0.0001**	**94.80**
Sex				
Male	1947	Reference		**94.47**
Female	1948	-0.4385 (-0.4466, -0.4304)	**<0.0001**	
Study year	3895	-0.0335 (-0.0617, -0.0052)	**<0.05**	**93.99**
Economic region				
East	1818	Reference		
Central	76	0.1568 (-0.3728, 0.6864)	0.5618	**93.27**
West	1072	0.0132 (-1.0939, 1.1203)	0.9813	
Northeast	592	-0.0352 (-0.4789, 0.4085)	0.8764	
Setting				
Urban	782	Reference		**93.91**
Rural	2221	-0.5670 (-0.6140, -0.5200)	**<0.0001**	
**PACG**				
Age	3765	0.0461 (0.0458, 0.0464)	**<0.0001**	**94.19**
Sex				
Male	1872	Reference		**93.78**
Female	1893	0.2116 (0.2039, 0.2192)	**<0.0001**	
Study year	3765	-0.0281 (-0.0527, -0.0035)	**<0.05**	**93.17**
Economic region				
East	1736	Reference		
Central	76	0.0353 (-0.8793, 0.9499)	0.9397	**91.53**
West	1052	-0.1369 (-0.5284, 0.2546)	0.4931	
Northeast	564	0.2487 (-0.2181, 0.7154)	0.2964	
Setting				
Urban	768	Reference		**96.16**
Rural	2123	0.4886 (0.4379, 0.5393)	**<0.0001**	

Notes: CI, confidence interval; POAG, primary open-angle glaucoma; PACG, primary angle-closure glaucoma.

Table S6. Multilevel mixed-effects meta-regression models of the national prevalence of primary open-angle glaucoma and primary angle-clousure glaucoma

Variable	Number of data points	*β* (95% CI)	*P*-value	I^2 (%)
**POAG**				
Intercept	3895	52.2846 (-5.5266 110.0958)	0.0763	**94.63**
Age	3895	0.0255 (0.0251, 0.0258)	**<0.0001**	
Sex				
Male	1947	Reference		
Female	1948	-0.4531 (-0.4612, -0.4451)	**<0.0001**	
Study year	3895	-0.0289 (-0.0576, -0.0001)	**<0.05**	
**PACG**				
Intercept	3765	50.8455 (1.5201, 100.1709)	**<0.05**	**93.94**
Age	3765	0.0459 (0.0456, 0.0462)	**<0.0001**	
Sex				
Male	1872	Reference		
Female	1893	0.1831 (0.1755, 0.1907)	**<0.0001**	
Study year	3765	-0.0261 (-0.0523, 0.0000)	0.0503	

Notes: CI, confidence interval; POAG, primary open-angle glaucoma; PACG, primary angle-closure glaucoma.

Table S7. Detailed characteristics of the included articles (n=43)

ID	Study	Study Year	Province	Setting	ACA/ depth evalu-ation	IOP	Optic disc evalu-ation	Visual field testing	Age range	Sam-ple	Female Propor-tion	POAG	PACG	Secondary glaucoma	Congenital glaucoma	Glaucoma
**S-01**	Xu L et al. (2004)	2001	Beijing	Mixed	Yes, suspects	Yes, all	Yes, all	Yes, all	40+	4451	0.564592226	81	99*	NA	NA	NA
**S-02**	Bai ZL et al. (2005)	2003	Shaanxi	Rural	Yes, all	Yes, all	Yes, all	Yes, suspects	40-91	2835	0.560493827	25*	31	NA	NA	NA
**S-03**	Xu L et al. (2005)	2001	Beijing	Mixed	Yes, suspects	Yes, all	Yes, all	Yes, all	40+	4431	0.565335139	51*	62	NA	NA	NA
**S-04**	Sun HM et al. (2005)	2003	Tianjin	Rural	Yes, all	Yes, all	Yes, all	Yes, suspects	40+	1701	0.501469724	6	19	2	0	27
**S-05**	Ren BC et al. (2005)	2003	Shaanxi	Rural	Yes, all	Yes, all	Yes, all	Yes, suspects	50-91	1775	0.544788732	7	29	2	0	38
**S-06**	He M et al. (2006)	2003	Guangdong	Urban	Yes, all	Yes, all	Yes, all	Yes, suspects	50-93	1405	0.563701068	29	21	2	NA	NA
**S-07**	Bai YQ et al. (2007)	2006	Jiangxi	Rural	Yes, all	Yes, suspects	Yes, all	Yes, suspects	50-87	5013	0.581687612	62*	76	NA	NA	NA
**S-08**	Yuan HP et al. (2007)	2004	Jilin	Rural	Yes, all	Yes, all	Yes, all	Yes, suspects	41-96	1139	0.527655838	24*	29	NA	NA	NA
**S-09**	Deng ZF et al. (2008)	2005*	Shandong	Rural	Yes, all	Yes, all	Yes, all	Yes, suspects	40+	1166	0.441680961	15	18*	NA	NA	NA
**S-10**	Song SF et al. (2009)	2005	Chongqing	Mixed	Yes, all	Yes, all	Yes, all	Yes, suspects	50+	5938	0.57696194	51	148	12	0	211
**S-11**	Zhao X et al. (2010)	2006	Beijing	Urban	Yes, all	Yes, all	Yes, all	Yes, suspects	50+	2410	0.528215768	15	40	2	0	57
**S-12**	Zhang LJ et al. (2010)	2009	Inner Mongolia	Rural	Yes, all	Yes, all	Yes, all	Yes, suspects	40+	579	0.497409326	10	13	NA	NA	NA
**S-13**	Wang YX et al. (2010)	2001	Beijing	Mixed	Yes, all	Yes, all	Yes, all	Yes, all	40-101	4315	0.562224797	111	44	3	NA	NA

ID	Study	Study Year	Province	Setting	ACA/ depth evalu-ation	IOP	Optic disc evalu-ation	Visual field testing	Age range	Sam-ple	Female Propor-tion	POAG	PACG	Secondary glaucoma	Congenital glaucoma	Glaucoma
**S-14**	Liang YB et al. (2011)	2007	Hebei	Rural	Yes, all	Yes, all	Yes, all	Yes, suspects	30+	6716	0.535884455	125	153*	NA	NA	NA
**S-15**	Liang Y et al. (2011)	2007	Hebei	Rural	Yes, all	Yes, all	Yes, all	Yes, suspects	30+	6716	0.535884455	42*	51	NA	NA	NA
**S-16**	Qu W et al. (2011)	2007	Heilongjiang	Rural	Yes, all	Yes, all	Yes, all	Yes, suspects	40+	4956	0.550443906	64*	78	NA	NA	NA
**S-17**	Song W et al. (2011)	2009	Inner Mongolia	Rural	Yes, all	Yes, all	Yes, all	Yes, suspects	40-87	5158	0.554284606	73	90	6	NA	NA
**S-18**	Sun J et al. (2012)	2007	Heilongjiang	Rural	Yes, all	Yes, all	Yes, all	Yes, suspects	40+	4956	0.649313963	35	43*	NA	NA	NA
**S-19**	Zhong H et al. (2012)	2010	Yunnan	Rural	Yes, all	Yes, all	Yes, all	Yes, suspects	50+	2133	0.639474918	22	20	5	NA	NA
**S-20**	Yu YY et al. (2013)	2010*	Heilongjiang	Rural	Yes, all	Yes, all	Yes, all	Yes, suspects	40+	2056	0.539883268	27*	33	NA	NA	NA
**S-21**	Sheng WD (2014)	2013	Inner Mongolia	Mixed	Yes, all	Yes, all	Yes, all	Yes, suspects	50-93	1068	0.54588015	13*	16	NA	NA	NA
**S-22**	Yin LR et al. (2014)	2010	Beijing	Urban	Yes, all	Yes, all	Yes, all	Yes, all	40-80	13016	0.626997541	136	110	NA	NA	NA
**S-23**	Gao ZZ et al. (2015)	2011	Jilin	Urban	Yes, all	Yes, all	Yes, all	Yes, suspects	40+	2359	0.431114879	33	48	NA	NA	NA
**S-24**	Pan YJ (2015)	2014	Guangdong	Urban	Yes, all	Yes, all	Yes, all	Yes, suspects	55-96	2422	NA	46	37	3	NA	NA
**S-25**	He J et al. (2015)	2011	Shanghai	Urban	Yes, all	Yes, all	Yes, all	Yes, suspects	50-106	2528	0.577531646	72	88*	NA	NA	NA
**S-26**	Pan CW et al. (2016)	2010	Yunnan	Rural	Yes, all	Yes, all	Yes, all	Yes, suspects	50+	6546	0.602352582	137	46	29	NA	NA

ID	Study	Study Year	Province	Setting	ACA/ depth evalu-ation	IOP	Optic disc evalu-ation	Visual field testing	Age range	Sam-ple	Female Propor-tion	POAG	PACG	Secondary glaucoma	Congenital glaucoma	Glaucoma
**S-27**	Cai Ji-wu et al. (2018)	2017	Guangdong	Rural	No	Yes, all	Yes, all	No	52-87	1200	0.465833333	5	25	2	NA	NA
**S-28**	ZHU Dan et al. (2018)	2015*	Liaoning	Urban	Yes, all	Yes, all	Yes, all	Yes, suspects	40+	4718	0.431114879	66	96	NA	NA	NA
**S-29**	ZHANG Min et al. (2018)	2017	Hebei	Mixed	Yes, all	Yes, all	Yes, all	Yes, all	5m-86	4960	0.486895161	27	70	23	15	135
**S-30**	Jonas J.B. et al. (2018)	2011	Beijing	Mixed	Yes, all	Yes, all	Yes, all	No	50-93	3127	0.565398145	91	41	NA	NA	NA
**S-31**	ZHOU Wei, et al. (2018)	2016	Tianjin	Mixed	Yes, all	Yes, all	Yes, all	Yes, all	50+	4048	0.60770751	65*	80	NA	NA	NA
**S-32**	Hai-ming Xu, et al. (2018)	2015	Zhejiang	Urban	Yes, all	Yes, all	Yes, all	Yes, all	50-88	2363	0.531527719	21*	25*	NA	NA	52
**S-33**	WU Xiao-lan et al. (2019)	2015	Zhejiang	Mixed	No	Yes, all	Yes, all	No	50-106	5448	NA	23*	29*	NA	NA	59
**S-34**	LI Tai-dong, et al. (2019)	2018	Sichuan	Mixed	No	Yes, all	Yes, all	No	36-95	2654	0.474001507	42*	51*	NA	NA	105
**S-35**	LI Wei-wei, et al. (2019)	2018	Zhejiang	Mixed	No	Yes, all	Yes, all	No	50+	1488	NA	7*	8*	NA	NA	17
**S-36**	LIU Yan-jun (2020)	2019	Sichuan	Urban	Yes, all	Yes, all	Yes, all	No	50+	4190	0.543914081	18*	23*	NA	NA	46
**S-37**	HUANG Bo-shu (2020)	2018	Jiangxi	Rural	Yes, all	Yes, all	Yes, all	Yes, all	40+	5385	0.587372331	31*	38*	NA	NA	78
**S-38**	CHENG hong et al. (2021)	2017	Anhui	Mixed	Yes, all	Yes, all	Yes, all	Yes, suspects	50+	550	NA	3	5	2	NA	NA
**S-39**	Yang, X., et al. (2021)	2018	Liaoning, Shandong and Jiangsu, Heilongjiang, Henan,	Rural	Yes, all	Yes, all	Yes, all	Yes, suspects	40+	33701	0.606866265	247	328	138	NA	NA

ID	Study	Study Year	Province	Setting	ACA/ depth evalu-ation	IOP	Optic disc evalu-ation	Visual field testing	Age range	Sam-ple	Female Propor-tion	POAG	PACG	Secondary glaucoma	Congenital glaucoma	Glaucoma
**S-40**	Yan, X., et al. (2021)	2018	Shaanxi and Shanxi, Ningxia Hui Autonomous Region, Sichuan and Chongqing Shandong, Jiangsu, Ningxia, Shaanxi, Sichuan, Chongqing, Shanxi, Heilong jiang, Liaoning and Henan	Rural	Yes, all	Yes, all	Yes, all	No	18+	28787	0.588	150*	184*	NA	NA	376
**S-41**	Yuanyuan, L., et al. (2022)	2018	Shandong and Jiangsu Provinces, Shanxi Province, Heilongjiang and Liaoning Provinces, Ningxia Hui Autonomous Region and Shaanxi Province, Henan Province, Sichuan Province and Chongqing Municipality	Rural	Yes, all	Yes, all	Yes, all	Yes, suspects	6+	48398	0.577337907	279	357	147	21	804
**S-42**	Zuo-xin Qin (2023)	2018	Chongqing	Rural	Yes, all	Yes, all	Yes, all	Yes, suspects	40+	4073	0.636876995	27	29	14	NA	NA

ID	Study	Study Year	Province	Setting	ACA/ depth evalu-ation	IOP	Optic disc evalu-ation	Visual field testing	Age range	Sam-ple	Female Propor-tion	POAG	PACG	Secondary glaucoma	Congenital glaucoma	Glaucoma
**S-43**	Zhang, A., et al (2023)	2018	Shandong and Jiangsu Provinces, Shanxi Province, Heilongjiang and Liaoning Provinces, Ningxia Hui Autonomous Region and Shaanxi Province, Henan Province, Sichuan Province and Chongqing Municipality	Rural	Yes, all	Yes, all	Yes, all	Yes, suspects	40+	33699	0.607317725	244	326	NA	NA	NA

Notes: NA, not available; ACA, Anterior chamber angle; IOP, intraocular pressure; POAG, primary open-angle glaucoma; PACG, primary angle-closure glaucoma. * indicates imputed study year and case number.

Table S8. Quality scores for assessing the risk of bias in the included articles (n=43)

Article ID	Study	Sample population	Sample size	Participation	Outcome assessment	Analytical methods	Total scores
**S-01**	Xu L et al. (2004)	2	1	1	2	2	8
**S-02**	Bai ZL et al. (2005)	2	1	1	2	2	8
**S-03**	Xu L et al. (2005)	2	1	1	2	2	8
**S-04**	Sun HM et al. (2005)	2	1	2	2	2	9
**S-05**	Ren BC et al. (2005)	2	2	1	2	2	9
**S-06**	He M et al. (2006)	2	1	1	2	2	8
**S-07**	Bai YQ et al. (2007)	2	1	2	2	2	9
**S-08**	Yuan HP et al. (2007)	2	1	1	2	2	8
**S-09**	Deng ZF et al. (2008)	2	1	2	2	2	9
**S-10**	Song SF et al. (2009)	2	2	2	2	2	10
**S-11**	Zhao X et al. (2010)	2	1	2	2	2	9
**S-12**	Zhang LJ et al. (2010)	2	1	2	2	2	9
**S-13**	Wang YX et al. (2010)	2	1	1	2	2	8
**S-14**	Liang YB et al. (2011)	2	1	0	2	2	7
**S-15**	Liang Y et al. (2011)	2	1	1	2	2	8
**S-16**	Qu W et al. (2011)	2	1	2	2	2	9
**S-17**	Song W et al. (2011)	2	1	2	2	2	9
**S-18**	Sun J et al. (2012)	2	1	2	2	2	9
**S-19**	Zhong H et al. (2012)	2	1	1	2	2	8
**S-20**	Yu YY et al. (2013)	2	1	2	2	2	9
**S-21**	Sheng WD (2014)	2	1	2	2	2	9
**S-22**	Yin LR et al. (2014)	2	1	0	2	2	7

Article ID	Study	Sample population	Sample size	Participation	Outcome assessment	Analytical methods	Total scores
**S-23**	Gao ZZ et al. (2015)	2	1	0	2	2	7
**S-24**	Pan YJ (2015)	2	2	2	2	2	10
**S-25**	He J et al. (2015)	2	2	1	2	2	9
**S-26**	Pan CW et al. (2016)	2	2	1	2	2	9
**S-27**	Cai Ji-wu et al. (2018)	2	0	2	2	2	8
**S-28**	ZHU Dan et al. (2018)	2	0	2	2	2	8
**S-29**	ZHANG Min et al. (2018)	2	0	2	2	2	8
**S-30**	Jonas J.B. et al. (2018)	2	1	1	2	2	8
**S-31**	ZHOU Wei, et al. (2018)	2	2	2	2	2	10
**S-32**	Hai-ming Xu, et al. (2018)	2	2	1	2	2	9
**S-33**	WU Xiao-lan et al. (2019)	2	2	2	2	2	10
**S-34**	LI Tai-dong, et al. (2019)	1	1	2	2	2	8
**S-35**	LI Wei-wei, et al. (2019)	2	1	2	2	2	9
**S-36**	LIU Yan-jun (2020)	2	0	2	1	2	7
**S-37**	HUANG Bo-shu (2020)	2	2	0	2	2	8
**S-38**	CHENG hong et al. (2021)	1	0	0	2	2	5
**S-39**	Yang, X., et al. (2021)	2	1	2	2	2	9
**S-40**	Yan, X., et al. (2021)	2	1	2	1	2	8
**S-41**	Yuanyuan, L., et al. (2022)	2	1	2	2	2	9
**S-42**	Zuo-xin Qin (2023)	2	2	2	2	2	10
**S-43**	Zhang, A., et al (2023)	2	1	2	2	2	9

Table S9. Main characteristics of the included articles (n=43)

Characteristics	N (%)
**Publish year**	
2000-2009	10 (23.3%)
2010-2019	25 (58.1%)
2019-2023	8 (18.6%)
**Economic region***	
East	19 (44.2%)
Central	3 (7.0%)
West	11 (25.6%)
Northeast	6 (14.0%)
**Study setting**	
Urban	9 (20.9%)
Rural	22 (51.2%)
Mixed	12 (27.9%)
**Subtype**	
POAG	27 (62.8%)
PACG	31 (72.1%)
Secondary glaucoma	16 (37.2%)
Congenital glaucoma	6 (14.0%)
**Anterior chamber angle/depth evaluation**	
Yes, all	37 (86.0%)
Yes, suspects	2 (4.6%)
No	4 (9.3%)
**IOP measurement**	
Yes, all	42 (97.7%)
Yes, suspects	1 (2.3%)
No	0 (0.0%)
**Optic disc evaluation**	
Yes, all	43 (100.0%)
Yes, suspects	0 (0.0%)
No	0 (0.0%)
**Visual field testing**	
Yes, all	8 (18.6%)
Yes, suspects	28 (18.6%)
No	7 (16.3%)
**Quality score**	
10	5 (11.6%)
9	18 (41.9%)
8	15 (34.9%)
7	4 (9.3%)
6	0 (0.0%)
5	1 (2.3%)

Notes: POAG, primary open-angle glaucoma; PACG, primary angle-closure glaucoma; IOP, intraocular pressure. *Four articles provided prevalene data based on multiple provinces.

Table S10. Estimated age- and sex-specific prevalence and case number of primary open-angle glaucoma by economic regions in the mainland of China in 2020

Age group	Case number (million, 95% CI)			Prevalence (%, 95% CI)		
**East China**						
	**Both**	**Male**	**Female**	**Both**	**Male**	**Female**
**20-29 year**	0.28 (0.20, 0.39)	0.18 (0.13, 0.25)	0.10 (0.07, 0.14)	0.39 (0.28, 0.56)	0.47 (0.33, 0.67)	0.30 (0.21, 0.42)
**30-39 year**	0.49 (0.34, 0.69)	0.31 (0.22, 0.44)	0.18 (0.13, 0.25)	0.49 (0.35, 0.69)	0.59 (0.42, 0.84)	0.38 (0.27, 0.53)
**40-49 year**	0.53 (0.37, 0.74)	0.33 (0.23, 0.46)	0.20 (0.14, 0.28)	0.64 (0.45, 0.91)	0.78 (0.55, 1.10)	0.50 (0.35, 0.70)
**50-59 year**	0.69 (0.49, 0.98)	0.43 (0.30, 0.60)	0.27 (0.19, 0.38)	0.81 (0.57, 1.14)	0.98 (0.70, 1.39)	0.63 (0.44, 0.89)
**60-69 year**	0.61 (0.43, 0.86)	0.37 (0.26, 0.52)	0.24 (0.17, 0.34)	1.04 (0.74, 1.47)	1.27 (0.90, 1.80)	0.82 (0.58, 1.15)
**70-79 year**	0.40 (0.28, 0.56)	0.24 (0.17, 0.33)	0.16 (0.11, 0.23)	1.31 (0.93, 1.85)	1.61 (1.14, 2.27)	1.03 (0.73, 1.45)
**80-89 year**	0.20 (0.14, 0.28)	0.11 (0.08, 0.15)	0.09 (0.07, 0.13)	1.63 (1.16, 2.30)	2.05 (1.45, 2.88)	1.32 (0.93, 1.86)
**90-99 year**	0.04 (0.03, 0.05)	0.02 (0.01, 0.02)	0.02 (0.01, 0.03)	1.95 (1.38, 2.73)	2.53 (1.80, 3.55)	1.64 (1.16, 2.30)
**20-99 year**	**3.23 (2.29, 4.56)**	**1.97 (1.40, 2.79)**	**1.26 (0.89, 1.78)**	**0.73 (0.52, 1.03)**	**0.88 (0.62, 1.24)**	**0.58 (0.41, 0.82)**
**Central China**						
**Age group**	**Case number (million, 95% CI)**			**Prevalence (%, 95% CI)**		
	**Both**	**Male**	**Female**	**Both**	**Male**	**Female**
**20-29 year**	0.15 (0.10, 0.21)	0.09 (0.07, 0.13)	0.05 (0.04, 0.08)	0.37 (0.26, 0.52)	0.45 (0.32, 0.63)	0.29 (0.20, 0.41)
**30-39 year**	0.25 (0.17, 0.35)	0.15 (0.11, 0.21)	0.10 (0.07, 0.14)	0.47 (0.33, 0.66)	0.57 (0.40, 0.80)	0.36 (0.26, 0.51)
**40-49 year**	0.31 (0.22, 0.44)	0.19 (0.14, 0.27)	0.12 (0.09, 0.17)	0.62 (0.44, 0.87)	0.75 (0.53, 1.06)	0.48 (0.34, 0.68)
**50-59 year**	0.45 (0.32, 0.64)	0.28 (0.19, 0.39)	0.18 (0.13, 0.25)	0.77 (0.55, 1.09)	0.95 (0.67, 1.34)	0.60 (0.43, 0.85)
**60-69 year**	0.38 (0.27, 0.53)	0.23 (0.16, 0.32)	0.15 (0.10, 0.21)	1.01 (0.71, 1.42)	1.23 (0.87, 1.73)	0.78 (0.55, 1.11)
**70-79 year**	0.28 (0.20, 0.39)	0.17 (0.12, 0.23)	0.11 (0.08, 0.16)	1.26 (0.89, 1.77)	1.54 (1.09, 2.17)	0.99 (0.70, 1.39)
**80-89 year**	0.13 (0.09, 0.18)	0.07 (0.05, 0.10)	0.06 (0.04, 0.08)	1.56 (1.11, 2.20)	1.96 (1.39, 2.75)	1.26 (0.89, 1.77)
**90-99 year**	0.02 (0.01, 0.03)	0.01 (0.01, 0.01)	0.01 (0.01, 0.02)	1.87 (1.33, 2.63)	2.44 (1.74, 3.43)	1.57 (1.11, 2.21)
**20-99 year**	**1.96 (1.39, 2.77)**	**1.19 (0.84, 1.67)**	**0.78 (0.55, 1.10)**	**0.72 (0.51, 1.02)**	**0.88 (0.62, 1.24)**	**0.57 (0.41, 0.81)**
**West China**						

Age group	Case number (million, 95% CI)			Prevalence (%, 95% CI)		
	Both	Male	Female	Both	Male	Female
**20-29 year**	0.17 (0.12, 0.24)	0.11 (0.08, 0.15)	0.06 (0.05, 0.09)	0.37 (0.26, 0.52)	0.44 (0.31, 0.63)	0.28 (0.20, 0.40)
**30-39 year**	0.26 (0.19, 0.37)	0.16 (0.12, 0.23)	0.10 (0.07, 0.14)	0.47 (0.33, 0.66)	0.57 (0.40, 0.80)	0.36 (0.26, 0.51)
**40-49 year**	0.35 (0.25, 0.50)	0.22 (0.16, 0.31)	0.13 (0.09, 0.19)	0.61 (0.43, 0.86)	0.74 (0.52, 1.05)	0.47 (0.34, 0.67)
**50-59 year**	0.46 (0.32, 0.64)	0.28 (0.20, 0.40)	0.18 (0.12, 0.25)	0.77 (0.55, 1.09)	0.94 (0.66, 1.32)	0.60 (0.42, 0.85)
**60-69 year**	0.37 (0.26, 0.52)	0.22 (0.16, 0.32)	0.14 (0.10, 0.20)	1.00 (0.71, 1.41)	1.22 (0.87, 1.72)	0.78 (0.55, 1.11)
**70-79 year**	0.28 (0.20, 0.39)	0.16 (0.12, 0.23)	0.11 (0.08, 0.16)	1.26 (0.89, 1.77)	1.55 (1.10, 2.18)	0.99 (0.70, 1.40)
**80-89 year**	0.13 (0.09, 0.18)	0.07 (0.05, 0.10)	0.06 (0.04, 0.08)	1.57 (1.12, 2.21)	1.96 (1.39, 2.76)	1.26 (0.89, 1.78)
**90-99 year**	0.02 (0.01, 0.03)	0.01 (0.01, 0.01)	0.01 (0.01, 0.01)	1.94 (1.37, 2.72)	2.46 (1.75, 3.46)	1.58 (1.12, 2.23)
**20-99 year**	**2.04 (1.44, 2.88)**	**1.24 (0.88, 1.75)**	**0.80 (0.56, 1.12)**	**0.71 (0.50, 1.00)**	**0.85 (0.60, 1.20)**	**0.56 (0.40, 0.79)**
**Northeast China**						
**Age group**	**Case number (million, 95% CI)**			**Prevalence (%, 95% CI)**		
	**Both**	**Male**	**Female**	**Both**	**Male**	**Female**
**20-29 year**	0.04 (0.03, 0.05)	0.02 (0.02, 0.03)	0.01 (0.01, 0.02)	0.38 (0.27, 0.54)	0.46 (0.33, 0.65)	0.29 (0.21, 0.42)
**30-39 year**	0.07 (0.05, 0.10)	0.04 (0.03, 0.06)	0.03 (0.02, 0.04)	0.49 (0.34, 0.69)	0.59 (0.42, 0.84)	0.38 (0.27, 0.53)
**40-49 year**	0.10 (0.07, 0.15)	0.06 (0.04, 0.09)	0.04 (0.03, 0.06)	0.63 (0.44, 0.89)	0.76 (0.54, 1.08)	0.49 (0.34, 0.69)
**50-59 year**	0.15 (0.11, 0.22)	0.09 (0.07, 0.13)	0.06 (0.04, 0.08)	0.80 (0.57, 1.13)	0.97 (0.69, 1.38)	0.62 (0.44, 0.88)
**60-69 year**	0.15 (0.11, 0.21)	0.09 (0.06, 0.13)	0.06 (0.04, 0.09)	1.03 (0.73, 1.45)	1.26 (0.90, 1.78)	0.81 (0.57, 1.14)
**70-79 year**	0.09 (0.06, 0.12)	0.05 (0.03, 0.07)	0.04 (0.03, 0.05)	1.31 (0.93, 1.84)	1.61 (1.14, 2.27)	1.04 (0.74, 1.47)
**80-89 year**	0.04 (0.03, 0.06)	0.02 (0.02, 0.03)	0.02 (0.01, 0.03)	1.68 (1.19, 2.37)	2.09 (1.49, 2.94)	1.36 (0.97, 1.92)
**90-99 year**	0.01 (0.01, 0.01)	0.00 (0.00, 0.01)	0.00 (0.00, 0.00)	2.07 (1.47, 2.91)	2.59 (1.84, 3.64)	1.69 (1.20, 2.37)
**20-99 year**	**0.65 (0.46, 0.91)**	**0.39 (0.27, 0.55)**	**0.26 (0.18, 0.37)**	**0.77 (0.55, 1.09)**	**0.93 (0.66, 1.32)**	**0.62 (0.44, 0.87)**

Notes: CI, confidence interval.

Table S11. Estimated provincial prevalence and case number of primary open-angle glaucoma in the mainland of China in 2020

Province	Case number (thousand, 95% CI)			Prevalence (%, 95% CI)		
	Both	Male	Female	Both	Male	Female
Beijing	147.89 (104.63, 208.83)	90.17 (63.82, 127.25)	57.72 (40.81, 81.57)	0.79 (0.56, 1.12)	0.95 (0.67, 1.34)	0.63 (0.45, 0.89)
Fujian	223.42 (158.05, 315.50)	136.56 (96.64, 192.74)	86.86 (61.40, 122.75)	0.71 (0.50, 1.00)	0.85 (0.60, 1.20)	0.56 (0.40, 0.79)
Guangdong	648.95 (458.99, 916.61)	406.02 (287.26, 573.22)	242.93 (171.73, 343.39)	0.68 (0.48, 0.96)	0.80 (0.57, 1.13)	0.54 (0.38, 0.76)
Jiangsu	527.23 (373.04, 744.31)	318.02 (225.10, 448.69)	209.21 (147.93, 295.62)	0.77 (0.55, 1.09)	0.93 (0.66, 1.31)	0.61 (0.43, 0.87)
Zhejiang	389.93 (275.86, 550.58)	242.03 (171.29, 341.56)	147.90 (104.57, 209.01)	0.73 (0.52, 1.04)	0.88 (0.62, 1.24)	0.58 (0.41, 0.82)
Tianjin	89.40 (63.25, 126.23)	54.78 (38.77, 77.29)	34.63 (24.48, 48.93)	0.79 (0.56, 1.11)	0.94 (0.67, 1.33)	0.62 (0.44, 0.88)
Shanghai	172.97 (122.38, 244.20)	106.00 (75.03, 149.58)	66.97 (47.35, 94.62)	0.80 (0.56, 1.12)	0.95 (0.67, 1.33)	0.64 (0.45, 0.90)
Shandong	577.78 (408.80, 815.70)	346.05 (244.94, 488.26)	231.73 (163.85, 327.44)	0.75 (0.53, 1.05)	0.90 (0.64, 1.27)	0.60 (0.42, 0.84)
Hebei	402.59 (284.83, 568.41)	242.01 (171.29, 341.49)	160.58 (113.54, 226.92)	0.73 (0.51, 1.02)	0.88 (0.62, 1.24)	0.57 (0.41, 0.81)
Hainan	51.37 (36.34, 72.54)	31.88 (22.56, 45.00)	19.48 (13.78, 27.54)	0.68 (0.48, 0.97)	0.81 (0.57, 1.14)	0.54 (0.39, 0.77)
Anhui	335.74 (237.55, 473.99)	202.85 (143.58, 286.20)	132.89 (93.97, 187.79)	0.73 (0.52, 1.03)	0.88 (0.62, 1.24)	0.58 (0.41, 0.82)
Jiangxi	229 (162.01, 323.34)	139.03 (98.40, 196.19)	89.97 (63.61, 127.15)	0.71 (0.50, 1.01)	0.86 (0.61, 1.21)	0.56 (0.40, 0.80)
Hunan	367.98 (260.36, 519.48)	223.76 (158.39, 315.69)	144.22 (101.97, 203.79)	0.74 (0.52, 1.05)	0.90 (0.63, 1.26)	0.59 (0.41, 0.83)
Hubei	338.05 (239.17, 477.27)	205.94 (145.77, 290.59)	132.11 (93.41, 186.68)	0.74 (0.52, 1.04)	0.89 (0.63, 1.25)	0.59 (0.41, 0.83)
Shanxi	195.41 (138.24, 275.92)	119.81 (84.79, 169.07)	75.60 (53.45, 106.84)	0.71 (0.50, 1.00)	0.86 (0.61, 1.21)	0.56 (0.40, 0.79)
Henan	497.13 (351.72, 701.89)	294.68 (208.57, 415.80)	202.45 (143.15, 286.09)	0.71 (0.50, 1.00)	0.86 (0.61, 1.22)	0.56 (0.40, 0.80)
Guizhou	181.50 (128.40, 256.28)	109.85 (77.74, 155.02)	71.65 (50.65, 101.26)	0.68 (0.48, 0.96)	0.82 (0.58, 1.15)	0.54 (0.38, 0.76)
Ningxia	35.97 (25.44, 50.80)	22.00 (15.57, 31.05)	13.97 (9.88, 19.75)	0.68 (0.48, 0.96)	0.82 (0.58, 1.16)	0.53 (0.38, 0.75)
Inner Mongolia	144.48 (102.21, 204.00)	88.25 (62.46, 124.54)	56.23 (39.75, 79.46)	0.73 (0.52, 1.04)	0.88 (0.62, 1.25)	0.58 (0.41, 0.82)
Qinghai	29.08 (20.57, 41.07)	17.88 (12.65, 25.24)	11.20 (7.92, 15.83)	0.67 (0.47, 0.95)	0.81 (0.57, 1.14)	0.53 (0.37, 0.75)
Shaanxi	220.99 (156.35, 312.03)	135.24 (95.72, 190.84)	85.75 (60.63, 121.19)	0.72 (0.51, 1.01)	0.86 (0.61, 1.22)	0.56 (0.40, 0.80)
Chongqing	194.03 (137.29, 273.91)	117.36 (83.08, 165.57)	76.67 (54.21, 108.34)	0.77 (0.55, 1.09)	0.93 (0.66, 1.31)	0.61 (0.43, 0.86)
Sichuan	487.23 (344.75, 687.80)	295.11 (208.90, 416.33)	192.12 (135.85, 271.47)	0.74 (0.52, 1.04)	0.89 (0.63, 1.26)	0.59 (0.41, 0.83)
Xinjiang	121.93 (86.25, 172.22)	75.74 (53.59, 106.93)	46.19 (32.65, 65.29)	0.66 (0.46, 0.93)	0.79 (0.56, 1.11)	0.51 (0.36, 0.73)

Province	Case number (thousand, 95% CI)			Prevalence (%, 95% CI)		
	Both	Male	Female	Both	Male	Female
Gansu	130.47 (92.30, 184.22)	79.60 (56.34, 112.33)	50.87 (35.96, 71.89)	0.69 (0.49, 0.98)	0.84 (0.59, 1.19)	0.54 (0.38, 0.77)
Yunnan	232.84 (164.71, 328.80)	143.38 (101.46, 202.36)	89.46 (63.25, 126.44)	0.67 (0.47, 0.94)	0.79 (0.56, 1.12)	0.53 (0.37, 0.75)
Tibet	14.23 (10.06, 20.10)	8.98 (6.35, 12.68)	5.25 (3.71, 7.42)	0.56 (0.40, 0.80)	0.67 (0.48, 0.95)	0.44 (0.31, 0.63)
Guangxi	246.34 (174.28, 347.80)	150.01 (106.17, 211.68)	96.33 (68.11, 136.12)	0.70 (0.50, 0.99)	0.84 (0.59, 1.18)	0.56 (0.40, 0.79)
Liaoning	287.04 (203.10, 405.21)	171.68 (121.53, 242.20)	115.36 (81.57, 163.00)	0.79 (0.56, 1.12)	0.96 (0.68, 1.35)	0.63 (0.45, 0.89)
Heilongjiang	207.53 (146.83, 292.99)	124.44 (88.08, 175.59)	83.09 (58.75, 117.41)	0.76 (0.54, 1.08)	0.92 (0.65, 1.30)	0.61 (0.43, 0.86)
Jilin	151.66 (107.30, 214.12)	90.7 (64.20, 127.98)	60.96 (43.10, 86.15)	0.75 (0.53, 1.06)	0.91 (0.64, 1.28)	0.60 (0.42, 0.85)

Notes: CI, confidence interval.

Table S12. Estimated age- and sex-specific prevalence and case number of primary angle-closure glaucoma by economic regions in the mainland of China in 2020

East China						
Age group	Case number (million, 95% CI)			Prevalence (%, 95% CI)		
	Both	Male	Female	Both	Male	Female
**20-29 year**	0.14 (0.11, 0.20)	0.07 (0.05, 0.10)	0.07 (0.05, 0.10)	0.20 (0.15, 0.28)	0.19 (0.14, 0.25)	0.23 (0.17, 0.31)
**30-39 year**	0.31 (0.23, 0.43)	0.15 (0.11, 0.20)	0.17 (0.12, 0.22)	0.32 (0.23, 0.43)	0.29 (0.21, 0.39)	0.35 (0.26, 0.47)
**40-49 year**	0.42 (0.31, 0.56)	0.19 (0.14, 0.26)	0.22 (0.16, 0.30)	0.51 (0.37, 0.69)	0.46 (0.34, 0.62)	0.56 (0.41, 0.76)
**50-59 year**	0.67 (0.50, 0.91)	0.31 (0.23, 0.42)	0.36 (0.27, 0.49)	0.79 (0.58, 1.06)	0.71 (0.53, 0.97)	0.86 (0.63, 1.16)
**60-69 year**	0.74 (0.55, 1.00)	0.33 (0.25, 0.45)	0.41 (0.30, 0.55)	1.26 (0.93, 1.70)	1.14 (0.84, 1.54)	1.37 (1.01, 1.85)
**70-79 year**	0.58 (0.43, 0.78)	0.25 (0.19, 0.34)	0.33 (0.24, 0.44)	1.91 (1.41, 2.57)	1.73 (1.28, 2.33)	2.08 (1.54, 2.80)
**80-89 year**	0.37 (0.28, 0.50)	0.14 (0.11, 0.19)	0.23 (0.17, 0.31)	3.04 (2.26, 4.08)	2.71 (2.01, 3.65)	3.29 (2.44, 4.41)
**90-99 year**	0.09 (0.07, 0.12)	0.03 (0.02, 0.04)	0.06 (0.05, 0.08)	4.52 (3.37, 6.04)	4.01 (2.98, 5.36)	4.80 (3.58, 6.40)
**20-99 year**	**3.33 (2.46, 4.50)**	**1.48 (1.09, 2.00)**	**1.85 (1.37, 2.50)**	**0.76 (0.56, 1.02)**	**0.66 (0.49, 0.89)**	**0.86 (0.63, 1.16)**
**Central China**						
Age group	Case number (million, 95% CI)			Prevalence (%, 95% CI)		
	Both	Male	Female	Both	Male	Female
**20-29 year**	0.09 (0.07, 0.12)	0.04 (0.03, 0.06)	0.05 (0.03, 0.06)	0.22 (0.16, 0.30)	0.20 (0.15, 0.28)	0.24 (0.18, 0.33)
**30-39 year**	0.18 (0.13, 0.25)	0.08 (0.06, 0.11)	0.10 (0.07, 0.13)	0.34 (0.25, 0.46)	0.31 (0.23, 0.42)	0.37 (0.27, 0.50)
**40-49 year**	0.28 (0.21, 0.38)	0.13 (0.10, 0.18)	0.15 (0.11, 0.21)	0.55 (0.41, 0.75)	0.51 (0.37, 0.68)	0.60 (0.45, 0.82)
**50-59 year**	0.50 (0.37, 0.67)	0.22 (0.17, 0.30)	0.27 (0.20, 0.37)	0.85 (0.63, 1.15)	0.77 (0.57, 1.04)	0.93 (0.68, 1.26)
**60-69 year**	0.51 (0.38, 0.70)	0.23 (0.17, 0.32)	0.28 (0.21, 0.38)	1.38 (1.02, 1.86)	1.25 (0.92, 1.69)	1.51 (1.11, 2.04)
**70-79 year**	0.46 (0.34, 0.62)	0.20 (0.15, 0.27)	0.26 (0.19, 0.35)	2.09 (1.55, 2.82)	1.89 (1.40, 2.55)	2.28 (1.69, 3.07)
**80-89 year**	0.27 (0.20, 0.36)	0.10 (0.08, 0.14)	0.17 (0.12, 0.22)	3.27 (2.43, 4.39)	2.92 (2.17, 3.92)	3.54 (2.63, 4.75)
**90-99 year**	0.05 (0.04, 0.07)	0.02 (0.01, 0.02)	0.04 (0.03, 0.05)	4.89 (3.65, 6.53)	4.28 (3.19, 5.73)	5.22 (3.90, 6.96)
**20-99 year**	**2.35 (1.74, 3.17)**	**1.04 (0.77, 1.40)**	**1.31 (0.97, 1.77)**	**0.87 (0.64, 1.17)**	**0.76 (0.57, 1.03)**	**0.97 (0.72, 1.31)**
**West China**						

Age group	Case number (million, 95% CI)			Prevalence (%, 95% CI)		
	Both	Male	Female	Both	Male	Female
**20-29 year**	0.11 (0.08, 0.14)	0.05 (0.04, 0.07)	0.06 (0.04, 0.08)	0.23 (0.17, 0.31)	0.21 (0.15, 0.28)	0.24 (0.18, 0.33)
**30-39 year**	0.19 (0.14, 0.26)	0.09 (0.07, 0.13)	0.10 (0.08, 0.14)	0.35 (0.26, 0.47)	0.32 (0.23, 0.43)	0.38 (0.28, 0.51)
**40-49 year**	0.33 (0.24, 0.44)	0.15 (0.11, 0.21)	0.17 (0.13, 0.24)	0.57 (0.42, 0.77)	0.52 (0.38, 0.70)	0.62 (0.46, 0.84)
**50-59 year**	0.50 (0.37, 0.68)	0.23 (0.17, 0.32)	0.27 (0.20, 0.37)	0.85 (0.63, 1.15)	0.78 (0.57, 1.05)	0.92 (0.68, 1.25)
**60-69 year**	0.51 (0.38, 0.69)	0.23 (0.17, 0.31)	0.28 (0.20, 0.37)	1.38 (1.02, 1.87)	1.26 (0.93, 1.70)	1.51 (1.11, 2.03)
**70-79 year**	0.46 (0.34, 0.62)	0.20 (0.15, 0.27)	0.26 (0.19, 0.35)	2.10 (1.56, 2.83)	1.90 (1.41, 2.57)	2.29 (1.69, 3.08)
**80-89 year**	0.26 (0.20, 0.36)	0.11 (0.08, 0.14)	0.16 (0.12, 0.21)	3.21 (2.39, 4.31)	2.88 (2.14, 3.87)	3.48 (2.59, 4.67)
**90-99 year**	0.05 (0.04, 0.07)	0.02 (0.01, 0.02)	0.03 (0.02, 0.04)	4.83 (3.60, 6.45)	4.30 (3.20, 5.75)	5.19 (3.87, 6.92)
**20-99 year**	**2.41 (1.79, 3.26)**	**1.08 (0.80, 1.47)**	**1.33 (0.98, 1.80)**	**0.84 (0.62, 1.13)**	**0.74 (0.55, 1.00)**	**0.94 (0.69, 1.26)**
**Northeast China**						
**Age group**	**Case number (million, 95% CI)**			**Prevalence (%, 95% CI)**		
	**Both**	**Male**	**Female**	**Both**	**Male**	**Female**
**20-29 year**	0.02 (0.01, 0.03)	0.01 (0.01, 0.01)	0.01 (0.01, 0.01)	0.21 (0.15, 0.29)	0.19 (0.14, 0.26)	0.23 (0.17, 0.31)
**30-39 year**	0.05 (0.04, 0.07)	0.02 (0.02, 0.03)	0.03 (0.02, 0.04)	0.33 (0.24, 0.44)	0.30 (0.22, 0.40)	0.36 (0.26, 0.48)
**40-49 year**	0.09 (0.06, 0.12)	0.04 (0.03, 0.06)	0.05 (0.03, 0.06)	0.53 (0.39, 0.72)	0.49 (0.36, 0.66)	0.58 (0.43, 0.79)
**50-59 year**	0.15 (0.11, 0.21)	0.07 (0.05, 0.10)	0.08 (0.06, 0.11)	0.81 (0.60, 1.10)	0.74 (0.54, 1.00)	0.88 (0.65, 1.19)
**60-69 year**	0.18 (0.14, 0.25)	0.08 (0.06, 0.11)	0.10 (0.08, 0.14)	1.26 (0.93, 1.70)	1.14 (0.84, 1.54)	1.37 (1.01, 1.85)
**70-79 year**	0.12 (0.09, 0.16)	0.05 (0.04, 0.07)	0.07 (0.05, 0.10)	1.87 (1.39, 2.52)	1.71 (1.26, 2.30)	2.01 (1.49, 2.71)
**80-89 year**	0.07 (0.05, 0.09)	0.03 (0.02, 0.04)	0.04 (0.03, 0.05)	2.78 (2.06, 3.73)	2.55 (1.89, 3.43)	2.95 (2.19, 3.96)
**90-99 year**	0.02 (0.01, 0.02)	0.01 (0.00, 0.01)	0.01 (0.01, 0.01)	4.26 (3.18, 5.69)	3.90 (2.90, 5.21)	4.53 (3.38, 6.04)
**20-99 year**	**0.70 (0.52, 0.94)**	**0.31 (0.23, 0.42)**	**0.39 (0.29, 0.53)**	**0.84 (0.62, 1.13)**	**0.74 (0.55, 1.00)**	**0.93 (0.69, 1.25)**

Notes: CI, confidence interval.

Table S13. Estimated provincial prevalence and case number of primary angle-closure glaucoma in the mainland of China in 2020

Province	Case number (thousand, 95% CI)			Prevalence (%, 95% CI)		
	Both	Male	Female	Both	Male	Female
Beijing	104.88 (77.52, 141.73)	46.49 (34.34, 62.87)	58.39 (43.17, 78.85)	0.56 (0.42, 0.76)	0.49 (0.36, 0.66)	0.64 (0.47, 0.86)
Fujian	234.21 (173.11, 316.48)	104.80 (77.42, 141.72)	129.41 (95.69, 174.76)	0.74 (0.55, 1.00)	0.65 (0.48, 0.88)	0.84 (0.62, 1.13)
Guangdong	599.33 (442.88, 810.08)	272.40 (201.18, 368.49)	326.92 (241.71, 441.59)	0.62 (0.46, 0.84)	0.54 (0.40, 0.73)	0.72 (0.53, 0.97)
Jiangsu	557.81 (412.47, 753.31)	243.89 (180.23, 329.65)	313.91 (232.23, 423.66)	0.82 (0.60, 1.10)	0.71 (0.53, 0.96)	0.92 (0.68, 1.24)
Zhejiang	402.85 (297.80, 544.25)	183.70 (135.72, 248.35)	219.15 (162.08, 295.89)	0.76 (0.56, 1.03)	0.67 (0.49, 0.90)	0.86 (0.64, 1.16)
Tianjin	77.14 (57.02, 104.23)	34.55 (25.53, 46.72)	42.59 (31.49, 57.51)	0.68 (0.50, 0.92)	0.59 (0.44, 0.80)	0.77 (0.57, 1.04)
Shanghai	142.54 (105.38, 192.52)	63.82 (47.15, 86.28)	78.72 (58.23, 106.24)	0.66 (0.49, 0.89)	0.57 (0.42, 0.77)	0.75 (0.55, 1.01)
Shandong	681.48 (503.86, 920.46)	294.63 (217.70, 398.28)	386.86 (286.16, 522.18)	0.88 (0.65, 1.19)	0.77 (0.57, 1.04)	0.99 (0.74, 1.34)
Hebei	471.89 (348.81, 637.59)	206.83 (152.80, 279.65)	265.06 (196.01, 357.94)	0.85 (0.63, 1.15)	0.75 (0.55, 1.01)	0.95 (0.70, 1.28)
Hainan	57.05 (42.17, 77.07)	25.76 (19.03, 34.83)	31.29 (23.15, 42.24)	0.76 (0.56, 1.03)	0.65 (0.48, 0.88)	0.87 (0.65, 1.18)
Anhui	409.80 (303.01, 553.45)	181.92 (134.44, 245.88)	227.88 (168.57, 307.57)	0.89 (0.66, 1.20)	0.79 (0.58, 1.07)	0.99 (0.73, 1.34)
Jiangxi	264.36 (195.41, 357.18)	117.22 (86.60, 158.49)	147.14 (108.81, 198.69)	0.82 (0.61, 1.11)	0.72 (0.53, 0.98)	0.92 (0.68, 1.24)
Hunan	443.92 (328.21, 599.59)	198.23 (146.48, 267.94)	245.69 (181.73, 331.65)	0.89 (0.66, 1.21)	0.79 (0.59, 1.07)	1.00 (0.74, 1.35)
Hubei	386.16 (285.46, 521.71)	172.86 (127.71, 233.70)	213.30 (157.75, 288.00)	0.84 (0.62, 1.14)	0.75 (0.55, 1.01)	0.95 (0.70, 1.28)
Shanxi	222.97 (164.80, 301.30)	101.09 (74.68, 136.69)	121.88 (90.12, 164.61)	0.81 (0.60, 1.10)	0.72 (0.53, 0.98)	0.90 (0.67, 1.22)
Henan	621.63 (459.58, 839.69)	264.80 (195.65, 357.98)	356.83 (263.93, 481.71)	0.89 (0.66, 1.20)	0.78 (0.57, 1.05)	0.99 (0.73, 1.34)
Guizhou	224.51 (165.95, 303.35)	99.48 (73.49, 134.51)	125.03 (92.46, 168.84)	0.84 (0.62, 1.13)	0.74 (0.55, 1.00)	0.94 (0.69, 1.27)
Ningxia	37.08 (27.39, 50.13)	16.87 (12.46, 22.82)	20.20 (14.93, 27.30)	0.70 (0.52, 0.95)	0.63 (0.46, 0.85)	0.77 (0.57, 1.04)
Inner Mongolia	155.33 (114.79, 209.92)	70.43 (52.02, 95.24)	84.90 (62.77, 114.68)	0.79 (0.58, 1.07)	0.70 (0.52, 0.95)	0.88 (0.65, 1.19)
Qinghai	29.92 (22.10, 40.46)	13.52 (9.98, 18.29)	16.40 (12.12, 22.17)	0.69 (0.51, 0.93)	0.61 (0.45, 0.83)	0.77 (0.57, 1.05)
Shaanxi	250.10 (184.86, 337.95)	112.59 (83.18, 152.24)	137.51 (101.68, 185.72)	0.81 (0.60, 1.09)	0.72 (0.53, 0.97)	0.90 (0.67, 1.22)
Chongqing	216.16 (159.85, 291.92)	97.25 (71.88, 131.43)	118.91 (87.97, 160.49)	0.86 (0.63, 1.16)	0.77 (0.57, 1.04)	0.94 (0.70, 1.28)
Sichuan	607.84 (449.46, 820.89)	272.52 (201.40, 368.29)	335.33 (248.06, 452.59)	0.92 (0.68, 1.25)	0.82 (0.61, 1.11)	1.02 (0.76, 1.38)
Xinjiang	124.51 (91.97, 168.39)	57.52 (42.47, 77.83)	66.99 (49.50, 90.56)	0.67 (0.49, 0.90)	0.60 (0.44, 0.81)	0.75 (0.55, 1.01)

Province	Case number (thousand, 95% CI)			Prevalence (%, 95% CI)		
	Both	Male	Female	Both	Male	Female
Gansu	161.85 (119.62, 218.72)	73.05 (53.97, 98.78)	88.79 (65.65, 119.94)	0.86 (0.63, 1.16)	0.77 (0.57, 1.04)	0.95 (0.70, 1.28)
Yunnan	284.01 (209.89, 383.84)	128.13 (94.63, 173.29)	155.88 (115.25, 210.55)	0.81 (0.60, 1.10)	0.71 (0.52, 0.96)	0.92 (0.68, 1.24)
Tibet	17.50 (12.93, 23.68)	7.95 (5.87, 10.76)	9.56 (7.06, 12.92)	0.70 (0.51, 0.94)	0.60 (0.44, 0.81)	0.81 (0.60, 1.09)
Guangxi	305.96 (226.21, 413.26)	135.57 (100.16, 183.28)	170.39 (126.05, 229.97)	0.87 (0.64, 1.18)	0.76 (0.56, 1.02)	0.99 (0.73, 1.34)
Liaoning	300.74 (222.33, 406.26)	131.89 (97.45, 178.28)	168.85 (124.88, 227.98)	0.83 (0.61, 1.12)	0.74 (0.54, 1.00)	0.93 (0.68, 1.25)
Heilongjiang	224.71 (166.08, 303.64)	99.02 (73.15, 133.90)	125.68 (92.93, 169.75)	0.83 (0.61, 1.12)	0.73 (0.54, 0.99)	0.92 (0.68, 1.24)
Jilin	172.40 (127.42, 232.95)	76.06 (56.19, 102.85)	96.33 (71.23, 130.10)	0.85 (0.63, 1.15)	0.76 (0.56, 1.03)	0.95 (0.70, 1.28)

Notes: CI, confidence interval.

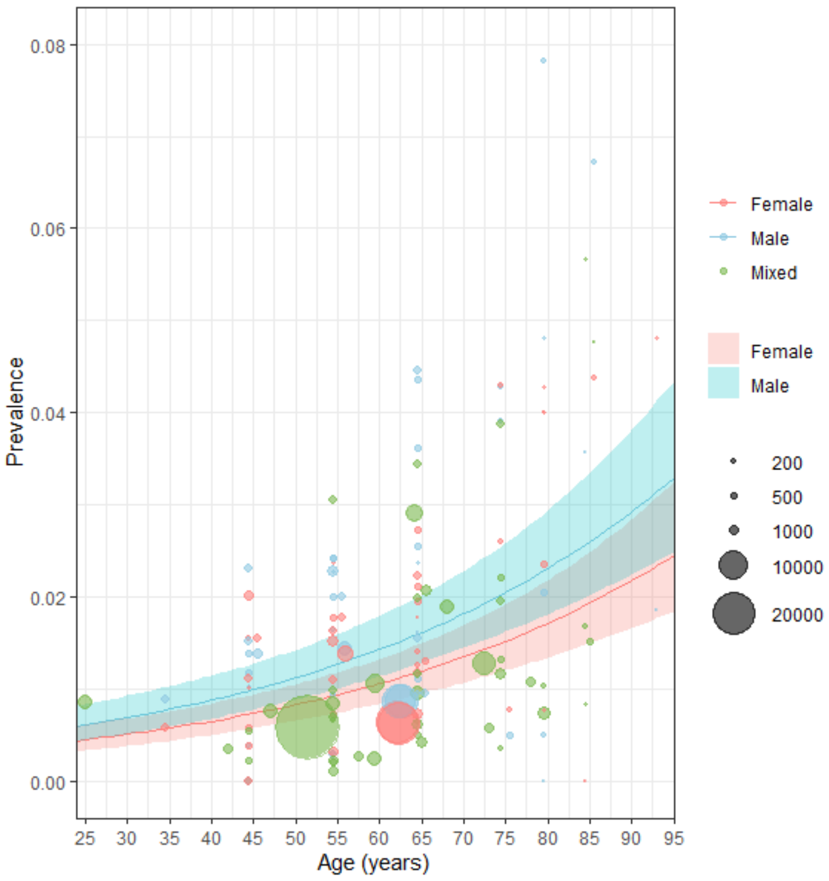

Figure S1. Rate pattern for primary open-angle glaucoma

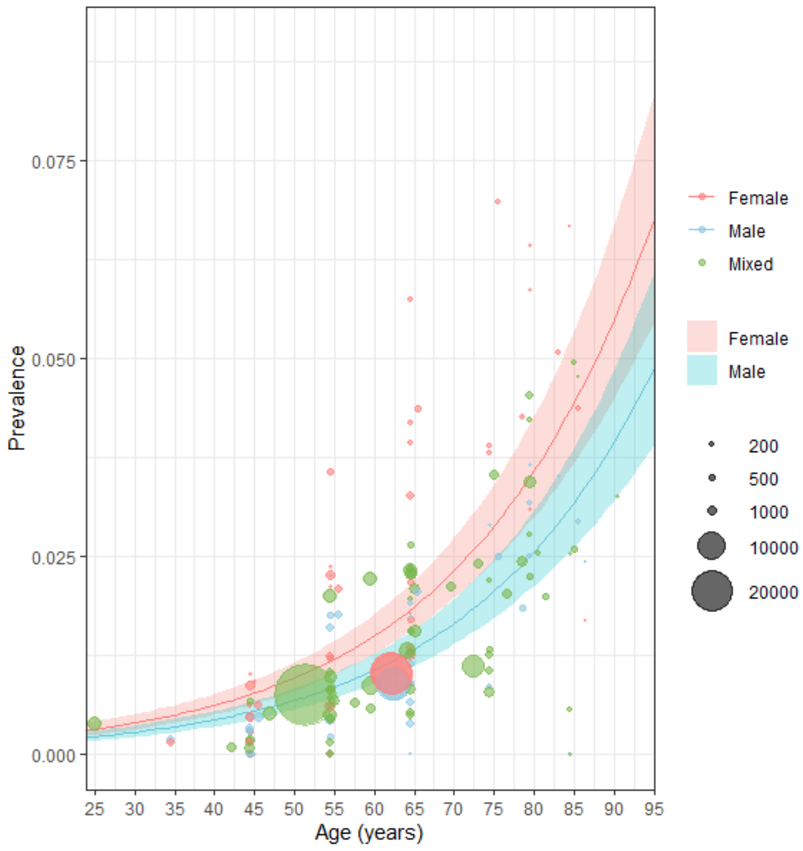

Figure S2. Rate pattern for primary angle-closure glaucoma

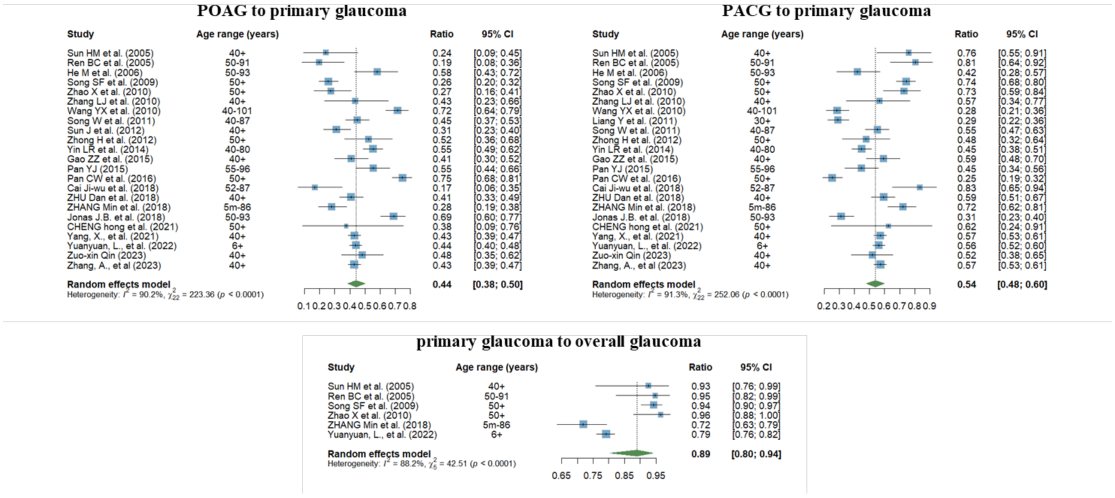

Figure S3. The meta-ratio of primary open-angle glaucoma to primary glaucoma, primary angle-closure glaucoma to primary glaucoma, and primary glaucoma to overall glaucoma

Notes: CI, confidence interval; POAG, primary open-angle glaucoma; PACG, primary angle-closure glaucoma.

Secondary glaucoma

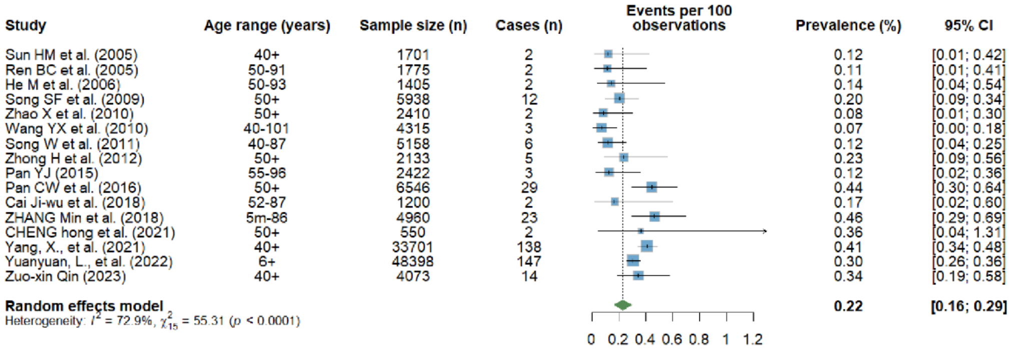

Congenital glaucoma

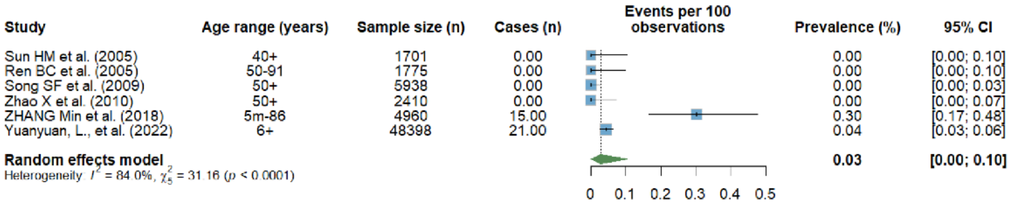

Figure S4. The pooled prevalence of secondary glaucoma and congenital glaucoma in the mainland of China.

Notes: CI, confidence interval.

Secondary glaucoma

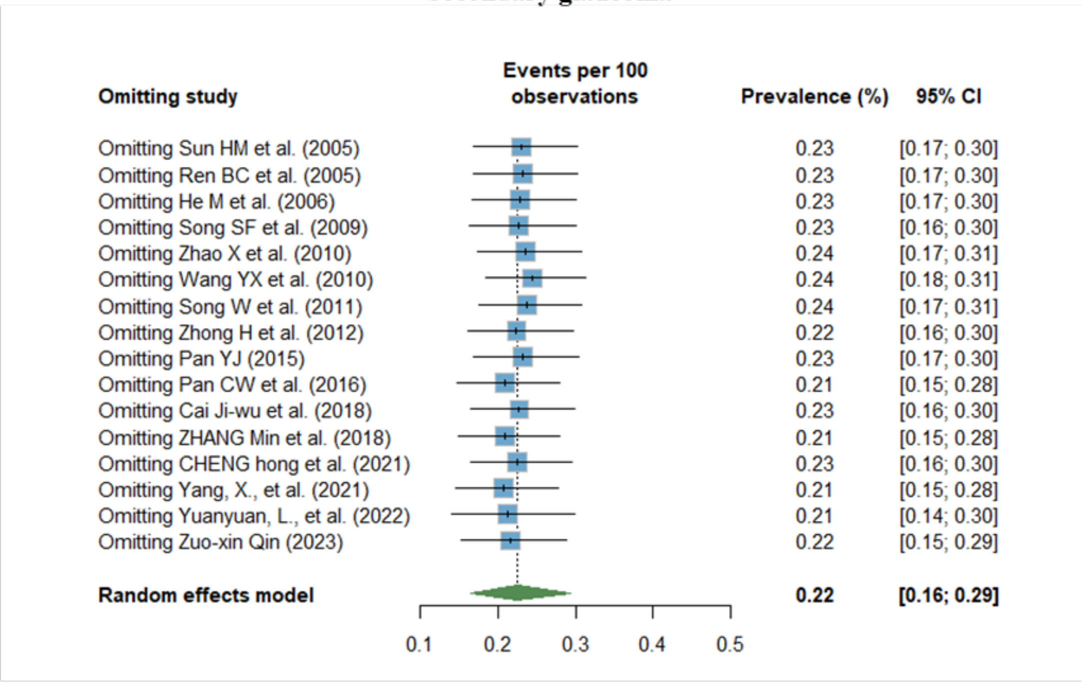

Congenital glaucoma

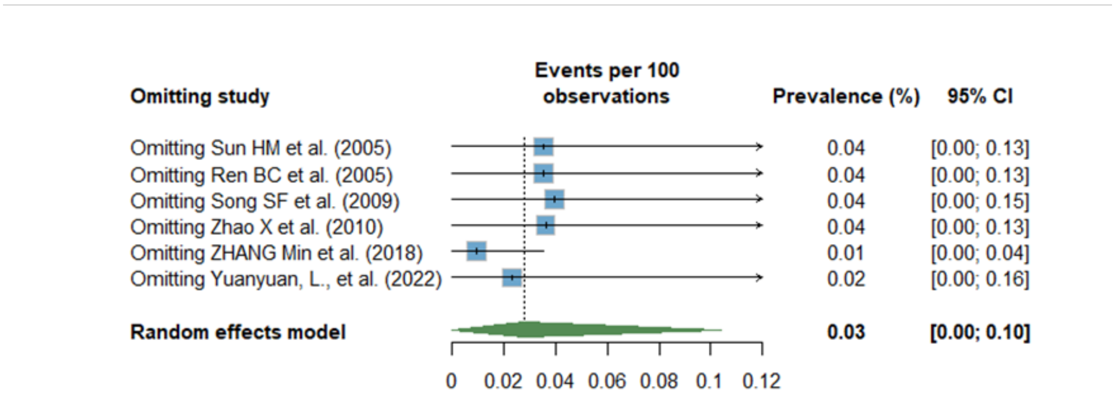

Figure S5. Leave-one-out sensitivity analysis for secondary glaucoma and congenital glaucoma

Notes: CI, confidence interval.

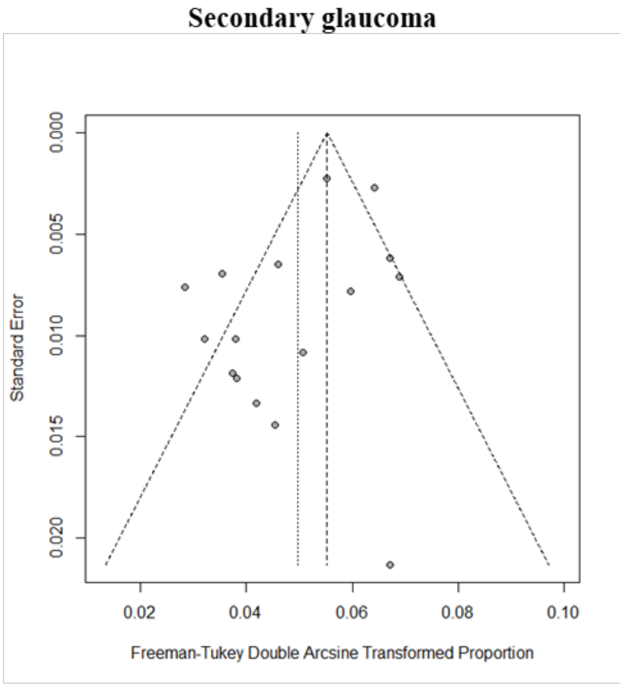

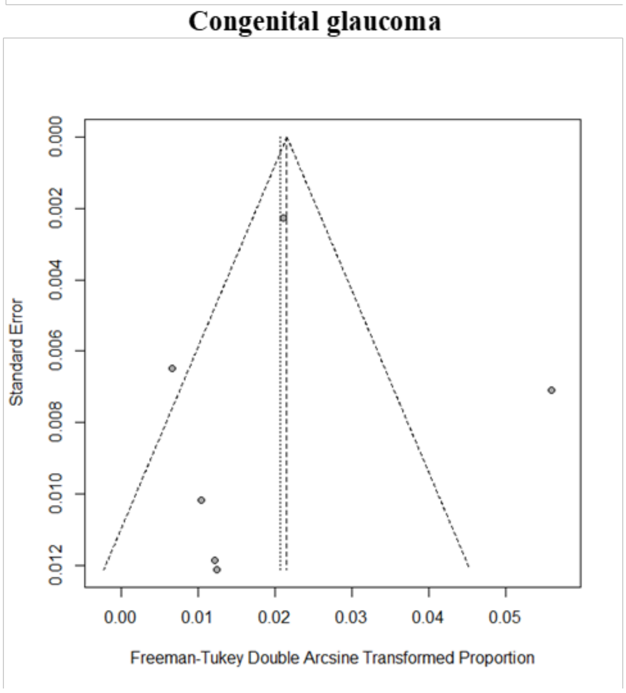

Figure S6. Funnel plots for the prevalence of secondary glaucoma and congenital glaucoma

Notes: CI, confidence interval. For secondary glaucoma, the results of Egger's test (*P*=0.0531) and Begg's test (*P*=0.5890) did not indicate significant publication bias.

Appendix 4. Full list of the included articles (n=43)

Article ID	Reference
**S-01**	Liang Xu, Jian-hua Chen, Jian-jun Li, et al. 徐亮, 陈建华, 李建军, et al. The prevalence and its screening methods of primary open angle glaucoma in defined population-based study of rural and urban in Beijing (北京农村及城市特定人群原发性开角型青光眼的患病率调查及其筛查方法评价)[J]. Chin J Ophthalmol (中华眼科杂志). 2004(11):9-15.
**S-02**	Lan-zhi Bai, Bai-chao Ren, Jian-gang Yang, et al. 白芝兰, 任百超, 杨建刚, 何媛, 陈莉, 孙乃学. Epidemiology of primary angle-closure glaucoma in a rural population in Shaanxi province of China (中国陕西省农村原发性闭角型青光眼流行病学调查)[J]. International journal of ophthalmology (国际眼科杂志). 2005;5(5):872-80.
**S-03**	Liang Xu, Li Zhang, Cui-ran Xia, et al. 徐亮, 张莉, 夏翠然, et al. The prevalence and its effective factors of primary angle-closure glaucoma in defined population of rural and urban in Beijing (北京农村及城市特定人群原发性闭角型青光眼的患病率及其影响因素)[J]. Chin J Ophthalmol (中华眼科杂志). 2005(01):12-8.
**S-04**	Hui-min Sun, Xiu-juan Zhang, Zhi-qing Li, et al. 孙慧敏, 张秀娟, 李志清, et al. Prevalence of glaucoma in Sangzi village, Ji county of Tianjin aged 40 years and above (天津市蓟县桑梓村 40 岁及以上人群中青光眼患病率调查)[J]. Chin J Pract Ophthalmol (中国实用眼科杂志). 2005(08):782-4.
**S-05**	Bai-chao Ren, Yuan He, Li Chen, et al. 任百超, 何媛, 陈莉, 杨建刚, 孙乃学. Epidemiology of glaucoma in a rural population in Shaanxi province (陕西省农村人群青光眼的流行病学调查)[J]. International journal of ophthalmology (国际眼科杂志). 2005(05):214-9.
**S-06**	He M, Foster PJ, Ge J, et al. Prevalence and clinical characteristics of glaucoma in adult Chinese: a population-based study in Liwan District, Guangzhou. Invest Ophthalmol Vis Sci 2006;47(7):2782-8.
**S-07**	Yong-quan Bai, Jing-lin Yi, Hui Xie, et al. 白永泉, 易敬林, 谢晖, et al. Epidemiological survey of primary angle-closure glaucoma in rural population aged 50 and elderly (吉安县农村 50 岁以上人群原发性闭角型青光眼流行病学调查)[J]. Medical Information section of operative surgery (医学信息(手术学分册)). 2007(09):774-7.
**S-08**	Hui-ping Yuan, Hong Yu, Zheng Xiao, et al. 原慧萍, 于泓, 肖铮, et al. The prevalence of primary angle-closure glaucoma and its causes in rural area of Shuangyang district in Changchun, Jilin province (吉林省长春市双阳区齐家乡原发性闭角青光眼的患病率调查及其影响因素)[J]. Chin J Ophthalmol (中华眼科杂志). 2007;43(9):775-8.
**S-09**	Zhi-feng Deng, Hong-juan Zhang. 邓志峰, 张洪娟. Epidemiology of primary open angle glaucoma in rural population in Heze (农村原发性开角型青光眼流行病学研究)[J]. Journal of Heze Medical College (菏泽医学专科学校学报). 2008(03):58-9.
**S-10**	Sheng-fang Song, Yong-ye Zhang, Xiang-ge He, et al. 宋胜仿, 张永烨, 贺翔鸽, et al. Prevalence of glaucoma among adults aged 50 years or above in Yongchuan district of Chongqing (重庆市永川地区 50 岁以上人群中青光眼患病率调查)[J]. Chin J Pract Ophthalmol (中国实用眼科杂志). 2009;27(2):168-72.
**S-11**	Xin Zhao, Yun-he He, Bi-qi Tian, et al. 赵欣, 郝云鹤, 田碧琪, et al. Glaucoma survey in population of 50 years old or more in the Xi Chang'an street community of Beijing (北京市西长安街社区 50 岁以上人群青光眼调查)[J]. Ophthalmol CHN (眼科). 2010(01):37-42.
**S-12**	Li-juan Zhang, Li Shan, Pan Fan, et al. 张丽娟, 单丽, 樊攀, 宋武莲, 原慧萍. Prevalence investigation on primary glaucoma in Kailu county, Inner Mongolia* (内蒙古开鲁县蒙古族原发性青光眼的患病率调查)[J]. Inner Mongolia Med J (内蒙古医学杂志). 2010(07):817-9.
**S-13**	Wang YX, Xu L, Yang H, Jonas JB. Prevalence of glaucoma in North China: the Beijing Eye Study. AM J OPHTHALMOL 2010;150(6):917-24.

Article ID	Reference
**S-14**	Liang YB, Friedman DS, Zhou Q, et al. Prevalence of primary open angle glaucoma in a rural adult Chinese population: the Handan eye study. Invest Ophthalmol Vis Sci 2011;52(11):8250-7.
**S-15**	Liang Y, Friedman DS, Zhou Q, et al. Prevalence and characteristics of primary angle-closure diseases in a rural adult Chinese population: the Handan Eye Study. Invest Ophthalmol Vis Sci 2011;52(12):8672-9.
**S-16**	Qu W, Li Y, Song W, et al. Prevalence and risk factors for angle-closure disease in a rural Northeast China population: a population-based survey in Bin County, Harbin. ACTA OPHTHALMOL 2011;89(6):e515-20.
**S-17**	Song W, Shan L, Cheng F, et al. Prevalence of glaucoma in a rural northern china adult population: a population-based survey in kailu county, inner mongolia. OPHTHALMOLOGY 2011;118(10):1982-8.
**S-18**	Sun J, Zhou X, Kang Y, et al. Prevalence and risk factors for primary open-angle glaucoma in a rural northeast China population: a population-based survey in Bin County, Harbin. Eye (Lond) 2012;26(2):283-91.
**S-19**	Zhong H, Li J, Li C, et al. The prevalence of glaucoma in adult rural Chinese populations of the Bai nationality in Dali: the Yunnan Minority Eye Study. Invest Ophthalmol Vis Sci 2012;53(6):3221-5.
**S-20**	Yang-yang Yu, Su-yun Wang, Shao-wei Wang. 于洋洋, 王素云, 王绍伟. Survey of prevalence of primary angle-closure glaucoma in rural population of Zhaozhou county (肇州县农村房角关闭疾病患病率调查)[J]. Chin J School Doctor (中国校医). 2013(08):599-601.
**S-21**	Wei-dong Sheng. 绳伟东. Epidemiological investigation on primary angle-closure glaucoma in middle-and-old aged people in Zhalantun city, Inner Mongolia* (内蒙古扎兰屯市中老年人群中原发性闭角型青光眼流行病学调查)[J]. China Prac Med (中国实用医药). 2014(24):261-3.
**S-22**	Lian-rong Yin, Hua Yang, Xin Li, et al. 尹连荣, 杨华, 李欣, 高健生. Primary discussion for education and screening on glaucoma in the western community in Beijing (京西社区青光眼宣教及筛查的初步探讨)[J]. Chinese Journal of Chinese Ophthalmology (中国中医眼科杂志). 2014(06):437-9.
**S-23**	Zhi-zhuo Gao, Tong Li, Yi-yuan Sun, et al. 高志卓, 李童, 孙艺源, et al. Epidemiological investigation of primary glaucoma in cold region of northern China (我国北方寒冷地区原发性青光眼流行病学调查分析)[J]. Chin J of Public Health Eng (中国卫生工程学). 2015(06):552-4.
**S-24**	Yu-jin Pan. 潘裕锦. Prevalence of primary angle-closure glaucoma: a over 55 years population based survey in Sijihuacheng community of Shenzhen (深圳市四季花城社区 55 岁以上人群原发性闭角型青光眼的流行病学调查)[D]. Jinan University (暨南大学), 2015.
**S-25**	He J, Zou H, Lee RK, et al. Prevalence and risk factors of primary open-angle glaucoma in a city of Eastern China: a population-based study in Pudong New District, Shanghai. BMC OPHTHALMOL 2015;15:134.
**S-26**	Pan CW, Zhao CH, Yu MB, et al. Prevalence, types and awareness of glaucoma in a multi-ethnic population in rural China: the Yunnan Minority Eye Study. Ophthalmic Physiol Opt 2016;36(6):664-70.
**S-27**	Cai Ji-wu, et al. 蔡吉梧 et al. A study on screening, early diagnosis and treatment of glaucoma in rural areas in the northern part of Zhongshan City* (中山市北部农村青光眼病筛查及早诊早治研究)[J]. Chinese Manipulation & Rehabilitation Medicine (按摩与康复医学). 2018. 9(16).
**S-28**	Zhu Dan. Nian Chaoxia & X.N. Chao. 朱丹, 年朝霞与 X.N. Chao. Epidemiological survey of primary glaucoma among community-dwelling people in Shenyang* (沈阳市社区居民原发性青光眼流行病学调查)[J]. Chinese Health Engineering (中国卫生工程学), 2018. 17(04): p. 535-536.
**S-29**	Min Zhang, et al. 张敏, et al. Survey and analysis on clinical prevalence of glaucoma in Baoding* (保定地区青光眼患病率现状调查分析)[J]. International Journal of Ophthalmology (国际眼科杂志), 2018. 18(10): pp. 1870-1873.
**S-30**	Jonas, J.B., et al., Cognitive Function and Ophthalmological Diseases: The Beijing Eye Study. Scientific reports, 2018. 8(1): p. 4816.
**S-31**	Zhou Wei. 周伟. The epidemiological investigation of visual impairment in Tianjin (天津市视力残疾的流行病学抽样调查)[D]. Tianjin medicine university(天津医科大学), 2018.

Article ID	Reference
**S-32**	Hai-ming Xu, et al. 徐海铭, et al. Epidemiological Investigation of Eye Diseases among Adults Aged 50 Years or Older in the Communities of Hangzhou in China(杭州市下城区 50 岁及以上人群眼病流行病学调查)[J]. Chinese Journal of Optometry Ophthalmology and Visual Science(中华眼视光学与视觉科学杂志), 2018. 20(6).
**S-33**	WU Xiao-lan, et al. 吴晓兰, et al. Epidemiological investigation of ophthalmopathy among adults aged 50 years or above in Ningbo (宁波地区 50 岁及以上人群眼病流行病学调查)[J]. Chinese Journal of General Practice (中华全科医学), 2019, 17(3): 491-495. doi: 10.16766/j.cnki.issn.1674-4152.000716
**S-34**	LI Tai-dong, et al. 李太东, et al. Analysis on gender comparison of 5 major eye dieases among faculty members in a university in southwest China (西南某高校教职工 5 种主要眼病的性别比较). Pre Med Trib (预防医学论坛) 2019. 25(11): p. 801-803.
**S-35**	LI Wei-wei, ZHANG Xin. 李潍薇, 张昕与 Z. Xin. Investigation and analysis of the etiology of low vision and blindness in people over 50 years of age in Taizhou Luqiao area* (台州路桥地区 50 岁以上人群低视力及盲的病因调查分析). Chinese rural medicine (中国乡村医药), 2020. 27(06): p 57-58.
**S-36**	L. Yanjun 刘艳君, Analysis of Eye Health Screening of People Over 50 Years Old in Communities (社区 50 岁以上人群眼健康筛查的结果分析). China Health Standard Management (中国卫生标准管理), 2020. 11(22).
**S-37**	Huang Bo-shu. 黄博抒. The analysis of glaucoma related factors and family relationship in rural residents of Jiangxi Province(江西省农村居民青光眼相关因素及家庭关系现状分析)[D]. Nanchang university(南昌大学), 2020.
**S-38**	Cheng hong, et al. 程红等. Epidemiological survey of glaucoma in people over 50 years of age in the Baohai community of Hefei City* (合肥市包河社区 50 岁以上人群青光眼流行病学调查)[J]. Chinese Science and Technology Journal Database (Full Text Version) Medicine and Health (中文科技期刊数据库（全文版）医药卫生), 2021(5).
**S-39**	Yang, X., et al., The association between long-term exposure to ambient fine particulate matter and glaucoma: A nation-wide epidemiological study among Chinese adults. International journal of hygiene and environmental health, 2021. 238: p. 113858.
**S-40**	Yan, X., et al., Health insurance enrollment and vision health in rural China: an epidemiological survey. BMC health services research, 2021. 21(1): p. 761.
**S-41**	Yuanyuan, L., et al., Glaucoma in rural China (the Rural Epidemiology for Glaucoma in China (REG-China)): a national cross-sectional study. The British journal of ophthalmology, 2022. 107(10).
**S-42**	Zuo-xin Qin. 覃佐欣. Blinding rate and associated epidemiology of glaucoma in Chongqing, China(重庆市青光眼致盲率的流行病学研究)[D]. Army medical university (中国人民解放军陆军军医大学), 2022.
**S-43**	Zhang, A., et al., Impacts of heatwaves and cold spells on glaucoma in rural China: a national cross-sectional study. Environmental science and pollution research international, 2023. 30(16): p. 47248-47261.

Notes: The Chinese publication list uses the official English names or abbreviations of the journals. English titles were obtained from the journals themselves or from literature databases (China National Knowledge Infrastructure, Wanfang, and China Science and Technology Journal Database). If an official English translation of a journal name is not available, a Pinyin title is used. If an English translation of a title is not available, we have translated the title, marked it with an asterisk (*), and highlighted it in green.
